# Phenomenological assessment of psychedelics induced experiences: Translation and validation of the German Challenging Experience Questionnaire (CEQ) and Ego-Dissolution Inventory (EDI)

**DOI:** 10.1371/journal.pone.0264927

**Published:** 2022-03-16

**Authors:** Katharina Dworatzyk, Tallulah Jansen, Timo Torsten Schmidt

**Affiliations:** 1 Institute of Cognitive Science, Universität Osnabrück, Osnabrück, Germany; 2 Department of Education and Psychology, Freie Universität Berlin, Berlin, Germany; Tufts, UNITED STATES

## Abstract

Several measures have been designed to assess subjective experiences induced by psychedelic substances or other mind-altering drugs as well as non-pharmacological methods. Recently, two self-report questionnaires have been introduced to measure acute adverse effects following psilocybin ingestion and the phenomenon of ego-dissolution associated with the use of psychedelics, respectively. The 26-item Challenging Experience Questionnaire assesses multiple facets of psilocybin induced experiences on seven subscales, whereas the 8-item Ego-Dissolution Inventory consists of a unidimensional scale. In the present study, these questionnaires were translated into German and their psychometric properties then evaluated in an online survey on psychedelics induced experiences. Confirmatory factor analysis suggested an acceptable fit of the 7-factor structure of the German Challenging Experience Questionnaire with overall good internal consistency for all subscales. The factor structure did not differ based on gender or prior struggle with a psychiatric disorder, furthering the evidence of internal validity. Correlations with the State-Trait-Anxiety Inventory and the Altered States of Consciousness Rating Scale demonstrated convergent validity. Confirmatory factor analysis did not support the hypothesized single-factor structure of the German Ego-Dissolution Inventory and exploratory factor analysis suggested an alternative factor structure, where only five items loaded onto a common factor that was interpreted as ego-dissolution. Internal consistency of this 5-item measure was high and correlation with selected items of the Mystical Experience Questionnaire and Altered States of Consciousness Rating Scale supported convergent validity. Translation and validation of these questionnaires contribute to the advancement of common standards in the psychological and neuroscientific study of altered states of consciousness.

## Introduction

The study of alterations of consciousness contributes to an understanding of the structure and faculties of human consciousness and to the endeavor to establish relationships between neurophysiological processes and corresponding phenomenological aspects. Often the distinction is made between the characterization of specific phenomenology or contents of states of consciousness and the definition of levels (or global states) of consciousness [1]. While the study of levels of consciousness focuses on the question how conscious a person is, the study of states of consciousness aims to characterize different states in terms of their phenomenological dimensions. Phenomenological dimensions are aspects of the subjective experience covering sensation, perception, attention, memory, emotion, cognitive and self-control, body image, and sense of self [2–5]. While the subjective experience of being conscious varies between individuals, the adult individual usually has an intuitive idea of what is subjectively experienced as normal. This state is commonly referred to as the normal waking consciousness. Deviations from the average state of consciousness along the phenomenological dimensions can occur spontaneously as in daydreaming, hypnagogic states, sleep and dream states or may be actively induced by, for instance, meditation, hypnosis, sensory deprivation, or psychoactive substances. The scientific study of such changes in consciousness, so-called altered states of consciousness (ASC), requires a valid assessment of their phenomenology, where standardized questionnaires can allow comparisons between the alterations induced by different induction methods or between different studies using the same induction method.

Over the past decades, a number of questionnaires have been designed with different purposes and different methodological backgrounds to assess the subjective experience during ASC [e.g. 6–16]. While some aim to identify a common phenomenological pattern to provide profiles of differently induced ASC, others focus on a specific phenomenon that is typically associated with a certain means of induction. To contribute to common standards in the field of ASC assessment, the present study was conducted to evaluate the psychometric properties of German versions of two recently introduced questionnaires, the Challenging Experience Questionnaire (CEQ) [15] and the Ego-Dissolution Inventory (EDI) [16].

The CEQ assesses multiple facets of challenging experiences, otherwise known as bad trips, occasionally presenting upon the use of psychedelics [15]. The 26-item CEQ is rated on a 6-point scale with the following response options: “none; not at all”, “so slight, cannot decide”, “slight”, “moderate”, “strong”, “extreme (more than ever before in my life and stronger than 4)”. Previous factor analysis confirmed the psychometric structure of seven factors interpreted as (1) Fear, (2) Grief, (3) Physical Distress, (4) Insanity, (5) Isolation, (6) Death, (7) Paranoia.

The EDI was introduced as an 8-item scale to quantify alterations in the sense of self [16], referred to as ego-dissolution. Ego-dissolution has been characterized as a phenomenon that is typically experienced during ASC induced by classic psychedelics and is phenomenologically related to both mystical experiences and psychotic states [16–19]. The EDI comprises eight items and is rated on a visual analog scale, ranging from 0 (“No, not more than usually”) to 100 (“Yes, entirely or completely”). Previous exploratory factor analysis (EFA) showed that all EDI items loaded strongly and exclusively onto a single factor.

In the present study, we investigated the psychometric structure, reliability, and validity of our German translations based on data of an anonymous online survey to provide robust instruments for the study of challenging experiences and ego-dissolution in German-speaking populations. The scope of our validation was organized in accordance with the original validation studies of the English versions. For the CEQ, we supplemented the original analysis with the assessment of the relationships between challenging experiences and state anxiety and the somewhat opposite experience of a pleasant/blissful state. For the validation of the EDI, we additionally examined relationships between ego-dissolution and related phenomena of altered self-awareness, disembodiment and impaired control and cognition.

## Materials and methods

Study design and data analysis followed the structure of the original validation studies of the CEQ [15] and the EDI [16] with slight adaptations as described. All materials were assessed and approved by the ethics committee of the University of Osnabrück (2017-07-13). Data were collected anonymously. The collection of IP addresses and the use of cookies was disabled to ensure participants’ complete anonymity. After informed consent was provided by clicking “next” on the introductory page of the survey, that presented information about the study and a consent form, participants were forwarded to the questionnaire.

### Translation

To achieve equivalence in regards to conceptual and linguistic consistency, a combination of back translation [20] and the committee approach [21, 22] was employed. Three independent mostly bilingual translators carried out the forward translation from the English into the German language. Translations were compared, and mismatches discussed by an expert committee with significant knowledge of concepts in ASC research. After consensus was reached, the forward translation was reviewed by an external expert in the field of ASC research. Back-translation was then performed by two independent translators with English as their native language. Translations were reconciled and compared with the original wording of the items, and adjustments discussed by the expert committee until agreement was reached. As a last step, both questionnaires underwent simple cognitive pretesting in which two volunteers provided feedback on ambiguities or difficulties of understanding. The final translations are provided as supporting information (see S1 & S2 Appendices).

### Participants

A total of 2116 survey participants were recruited via advertisement on the Altered States Database website (http://asdb.info/), e-mail invitation, flyers, weblinks and social media over a period of ten months. Inclusion criteria were defined as reading and writing fluency in German, at least 18 years of age, and complete information on at least one first-hand experience with a classic psychedelic, including psilocybin, lysergic acid diethylamide (LSD), dimethyltryptamine (DMT; also including ayahuasca), and mescaline, with cocaine or with alcohol.

### Survey structure

German versions of the CEQ and the EDI were applied in an online survey called Study on Phenomena of Hallucinogen Induced Experiences (SPHINX), which was hosted on the survey platform SurveyMonkey (https://de.surveymonkey.com/).

Participants answered questions regarding their age, gender, educational background, previous or current struggle with a psychiatric disorder, and previous use of psychoactive substances. Information about psychiatric disorders was based on self-reports and included the following response options in a multiple-choice format: none, anxiety disorder, post-traumatic stress disorder, depression, bipolar disorder, alcohol dependency, other substance abuse, eating disorder, obsessive-compulsive disorder, schizophrenia, attention deficit hyperactivity disorder, psychotic disorder. Participants were then given the opportunity to report on up to five different experiences: (1) a challenging/bad trip experience induced by psilocybin, (2a) the most intense experience induced by a classic psychedelic, (2b) a typical experience induced by a classic psychedelic, (2c) a typical experience induced by cocaine, (2d) a typical experience induced by alcohol. Retrospective assessment of subjective effects occasioned by these substances was realized using the CEQ in the first part of the survey and the EDI in the second part of the survey as well as additional items for validation purposes (see Measures). Respondents were also asked to provide information about substance and dose taken, age at the time of their reported challenging experience, their willingness to repeat the experience including the challenging portion, time elapsed between the drug experience and survey completion, and the subjective intensity of the experience. A comprehensive list of items included in this survey and the exact wording can be found in S3 Appendix.

### Measures

To test the construct validity of CEQ and EDI, we included a set of items taken from related questionnaires: the State-Trait Anxiety Inventory (STAI) that comprises two scales to assess episodic as well as dispositional anxiety [23, 24], the Altered States of Consciousness Rating Scale (5DASC) that is used to characterize differently induced ASC experiences and covers pleasant experiences of oneness as well as unpleasant effects such as separation from oneself and the world [10, 11], the 30-item Mystical Experience Questionnaire (MEQ30) that measures different aspects of mystical-type experiences [13, 14, 25], and the Phenomenology of Consciousness Inventory (PCI) [7, 8, 26] that was designed to quantify patterns of the subjective experience during mostly non-pharmacologically induced ASC.

Alongside the CEQ, we included five items of the state anxiety subscale (STAI-S) and four items of the Oceanic Boundlessness (OBN) subscale of the 5DASC as a measure of pleasant/blissful experiences. The EDI was presented intermixed with eight items relating to ego-inflation (EII), the experience of increased self-assurance [16]. Consistent with the original validation study, we included seven items of the MEQ30 measuring unitive experience. We further selected twenty-five items of the OBN and the Dread of Ego-Dissolution (DED) scale of the 5DASC [10] which, as shown by a more recent study [11], can also be analyzed as Experience of Unity, Disembodiment, Impaired Control and Cognition, and Anxiety. Finally, four items of the Altered Self-Awareness and the Altered Body Image subscales of the PCI were included as a joint measure of altered self-experience to explore their relationship with the EDI. All items were selected from the German versions of the respective questionnaires.

### Statistical analysis CEQ

#### Confirmatory factor analysis

We performed a confirmatory factor analysis (CFA) with the same settings as applied in the original English validation to confirm the factor structure revealed by Barrett et al. [15] using responses to the German translation of the CEQ that was applied in the present study. CFA is used in scale development to test the validity of a hypothesized dimensional structure of a scale, that is relationships between factors and items and relationships between factors.

Suitability of our data for factor analysis was assessed by investigating skew and kurtosis of all CEQ items. It was also investigated whether responses to items exhausted their full range. Additionally, suitability of the data was assessed using the Kaiser-Meyer-Olkin measure of sampling adequacy (KMO) and Bartlett’s test of sphericity. A confirmatory factor model was then fit to the data according to the 7-factor model of the English CEQ. Latent factors were set to be measured by their respective items with all other item loadings equal to zero. Factor loadings were standardized by setting the variance of each latent factor to one. Fit indices were estimated using maximum likelihood (ML) estimation. A combination of fit indices was used to assess the resulting model fit, including the comparative fit index (CFI) [27], the standardized root mean square residual (SRMR) [28], and the root mean square error of approximation (RMSEA) [29].

#### Factorial invariance analysis

To test whether our German set of items exhibits a similar or invariant factor structure between different groups, we replicated the factorial invariance analysis performed by Barret et al. [15]. It is hypothesized that a scale’s psychometric properties must be found identical across groups in sequential, increasingly restricted models to ensure accurate comparability of factor means. Measurement non-invariance suggests that a construct has a different structure or meaning to different groups so that the construct cannot be meaningfully tested or construed across groups [30].

We applied a series of multiple-group CFAs to our CEQ sample using the *cfa* function from the *lavaan* package in R [31] with ML estimation. Typically, measurement invariance is addressed at three levels: weak, strong, and strict measurement invariance corresponding to configural invariance, measurement invariance, and structural invariance of the model [32]. We added more constraints in each sequential model for parameters to be equal across groups and then tested whether model fit improved or degraded across sequential models [15]. When strict measurement invariance can be shown, the group differences on any item are due only to group differences on the common factors [32].

Proving a model to show strong or strict factorial invariance provides evidence that an instrument, here the German CEQ, is measuring constructs in a similar fashion in each compared group and therefore exhibits internal validity as part of construct validity. Model fit was assessed using a combination of change in the CFI, SRMR, and RMSEA. These fit indices have been shown through simulation to be sensitive to both measurement invariance and lack of measurement invariance at the three levels [33]. Decrease in CFI > 0.01, increase in RMSEA > 0.015, and increase in SRMR > 0.01 between levels of factorial invariance (i.e. change between modeling steps) indicates non-invariance [33].

There is various clinical evidence of the therapeutic influence of psilocybin on psychiatric disorders but also for other kinds of interactions between psychological disorders and psychedelic experiences [34–38], which highlights the importance of evaluating the CEQ in respective populations. Factorial invariance analysis was, therefore, conducted to specifically test whether the CEQ’s factor structure would sustain when tested within the group of participants who self-reported previous struggle with a psychiatric disorder. In addition to testing for two levels of previous struggle with a psychiatric disorder (having vs not having had previous struggle), factorial invariance was also tested separately for two levels of gender (male vs female).

#### Internal consistency and convergent validity

Reliability was estimated using Cronbach’s alpha [39], a standard measure to assess a scale’s internal consistency. As supplementary analysis to those provided by the original validation study, we determined measures of convergent validity. We assessed the relationships between the CEQ items and additionally included items from the STAI-S and 5DASC (as specified under Measures). Pearson correlations were calculated between CEQ subscale and total score, with the sum score of five STAI-S items, hypothesizing a positive correlation, as well as the mean score of four OBN (5DASC) items, hypothesizing a negative correlation.

### Statistical analysis EDI

#### Confirmatory factor analysis

To validate the proposed single-factor structure of the German EDI and EII, a model was defined based on results from the original validation study [16], comprising two independent factors. Loadings of EDI items onto the ego-dissolution factor and loadings of EII items onto the ego-inflation factor were specified and all other loadings set to zero. Factor loadings were standardized by setting the variance of each latent factor to one. Distributional properties of items were assessed prior to CFA and Bollen-Stine bootstrap correction (1000 samples) applied, where the Mardia test indicated multivariate non-normality. The model parameters were then estimated using ML estimation and the model fit evaluated using the same set of fit indices as for the CEQ.

As it was possible for participants to report on a variable number of drug experiences in the second part of the survey, the total EDI sample was split into four subsamples corresponding to the reported drug experience: intense and typical psychedelics induced experiences (Psy-Intense; Psy-Typical), typical cocaine induced experiences (Cocaine), and typical alcohol induced experiences (Alcohol). Multiple CFAs were then performed to investigate the psychometric structure of the German EDI separately in these subsamples to avoid systematic bias resulting from sample dependency.

#### Exploratory factor analysis

Model rejection was followed by EFA to examine the likely multi-factorial structure of the German EDI. Suitability of the data was assessed using KMO and Bartlett’s test of sphericity. To determine the number of factors to extract, results from the (revised) MAP test [40–42], parallel analysis with 1000 random draws [42, 43], and Cattell’s [44] scree plot criterion were considered. EFA was then performed for each subsample separately and for the total sample, using principal axis factoring and oblique promax rotation. Scales were derived based on factor loadings from the pattern matrix and mean item scores of items loading onto a common factor used for further analysis.

#### Internal consistency and construct validity

Internal consistency of EDI and EII was determined by Cronbach’s alpha [39]. Construct validity of the German EDI was assessed in the same manner as in the original validation study [16]. Convergent validity was assessed through correlations with the MEQ30 measure of unitive experience. Additionally, we explored the relationship with ASC scales measuring related phenomena: the 5DASC subscales OBN and DED, and in their alternative structure the Experience of Unity, Disembodiment, Impaired Control and Cognition, Anxiety subscales, were hypothesized to correlate positively with the EDI but not with the EII as they relate to changes in self-experience observed during psychedelic experiences. Similarly, we assumed that PCI items relating to altered self-experience would demonstrate a positive relationship with the EDI but not with the EII.

Consistent with the original study, discriminant validity was assessed based on the scale’s ability to depict the specific relationship between ego-dissolution and psychedelics induced experiences. If the EDI exclusively maps ego-dissolution, a phenomenon only typical of psychedelics induced experiences, the relationship of reported drug dose and intensity with ego-dissolution should be stronger than the relationship with ego-inflation in the psychedelics subsamples and no significant differences should be found for the cocaine and alcohol subsample. To investigate this aspect of the relationship, strength of correlation was compared by a 2-tailed t-test for dependent correlation coefficients [45] in each subsample separately. Provided that the subjective intensity of an experience is comparable across substances, it can also be hypothesized that the relationship of subjective intensity with ego-dissolution in the psychedelics subsamples is different from the relationship in the cocaine and alcohol subsample and does not differ between the psychedelics subsamples. Differences in the relationship across subsamples were tested by regressing intensity ratings on ego-dissolution or ego-inflation scores (MATLAB’s aoctool function) and comparing the slope of the regression lines (multiple comparison tests using Tukey’s HSD criterion). Since experiences induced by these substances differ in terms of their phenomenology, the EDI can provide a diagnostic measure to classify experiences based on their phenomenological assessment. It was tested whether the data would be separable into groups, corresponding to typical experiences induced by psychedelics, cocaine or alcohol experiences, by training a Support Vector Machine (SVM) classifier (5-fold cross-validation, linear kernel, standard normalization transformation) using ego-dissolution and ego-inflation scores.

The strength of all correlations was assessed by Spearman’s rho. The bias corrected and accelerated bootstrap method (1000 samples) was used to compute the 95% confidence intervals of correlation coefficients. On examining relationships between ego-dissolution or ego-inflation and other ASC scales, reported drug dose and intensity of the experience, multiple correlations were tested for statistical significance. To minimize the number of false positives, the Benjamini-Hochberg procedure [46] was applied for each sample with the false discovery rate set to 1% so that the critical values were .008 (Psy-Intense), .007 (Psy-Typical), .003 (Cocaine), and .003 (Alcohol). Statistical analysis was performed using MATLAB (Mathworks, Version 2019a), including the Statistics and Machine Learning Toolbox, and SPSS (IBM, Version 25), including AMOS Graphics.

## Results

### Results CEQ

#### Sample characteristics

A total of 668 participants took part in the first part of the survey and reported on psilocybin induced challenging experiences. Information from 340 participants who reported on a challenging experience induced by a psychedelic substance other than psilocybin was not further processed and participants who did not match the inclusion criteria, that is (1) reading and writing fluency in German, (2) at least 18 years of age at the time of survey completion, (3) having had a challenging experience after ingesting psilocybin mushrooms in the past and (4) age of between 18 and 70 years old at the time of the reported challenging experience, were fully excluded from all analysis. After free response comments provided at the end of the survey were inspected for violations against the general study participation requirements, n = 288 reports remained in the final sample. For the factorial invariance analysis, participants who had previously struggled with a psychiatric disorder (n = 156) were coded as 1, irrespective of the kind of disorder. Participants who had not experienced any struggle (n = 129) were coded as 0. Participants who reported their gender as female (n = 61) were coded as 1 and participants who reported their gender as male (n = 224) were coded as 0. Data of three participants who reported their gender as “other” were not included in the factorial invariance analysis (a subsample of n = 285 was used). This was done in order to test whether the CEQ’s factor structure would prove to be invariant within these groups. The report of sample characteristics for the CEQ validation sample is presented in Table 1.

**Table 1:** Sample characteristics for the CEQ and EDI validation sample.

	CEQ validation sample					EDI validation sample				
N	288					836				
Age										
Mean	27.6					26.3				
Standard deviation	8.1					8.2				
Gender										
Female	21.2%					20.8%				
Male	77.8%					79.1%				
Other	1.0%					0.1%				
Education										
No degree					0.7%	0.6%				
Left school at 16 (Haupt-/Mittlerer Schulabschluss)					25.7%	22.3%				
High school diploma (Fachhochschulreife/Abitur)					43.8%	49.2%				
University degree					27.8%	26.2%				
Doctorate					2.1%	1.8%				
Lifetime illicit drug use	LSD	Psilo	DMT	Mesca	Coca	LSD	Psilo	DMT	Mesca	Coca
Never	22.6%	0.4%	67.3%	83.0%	30.2%	18.5%	23.3%	71.4%	86.1%	42.1%
Once only	8.3%	10.4%	9.4%	6.6%	12.2%	12.7%	12.7%	9.5%	6.1%	12.1%
2–5 times	25.0%	35.4%	12.2%	6.9%	22.9%	27.3%	31.5%	10.9%	5.3%	17.6%
6–10 times	12.2%	23.6%	4.5%	1.7%	10.8%	11.6%	14.8%	2.6%	1.2%	7.2%
11–15 times	6.6%	11.5%	2.1%	0.7%	3.8%	9.5%	7.2%	1.4%	0.7%	3.2%
16–25 times	5.6%	6.6%	1.4%	0.4%	5.9%	6.1%	4.4%	1.4%	0.2%	5.1%
26–50 times	7.3%	6.3%	1.7%	0.0%	5.9%	6.8%	3.5%	1.8%	0.0%	3.3%
51–100 times	8.7%	2.4%	0.4%	0.4%	1.7%	4.3%	1.1%	0.6%	0.2%	4.5%
> 100 times	3.8%	3.5%	0.4%	0.0%	6.2%	3.2%	1.6%	0.4%	0.1%	4.6%
Don’t know	0.0%	0.0%	1.1%	0.4%	0.4%	0.0%	0.0%	0.0%	0.0%	0.1%
Have you struggled with any psychiatric disorder in the past?										
No	45.5%					61.1%				
Yes	54.9%					38.9%				
Would you repeat the experience, including the challenging portion?										
No	59.7%					-				
Yes	40.3%					-				

LSD, Lysergic acid diethylamide; Psilo, Psilcybin; DMT, N,N-dimethyltryptamine or ayahuasca; Mesca, Mescaline; Coca, Cocaine.

#### Factor structure and internal validity of the German CEQ

**Fig 1:**
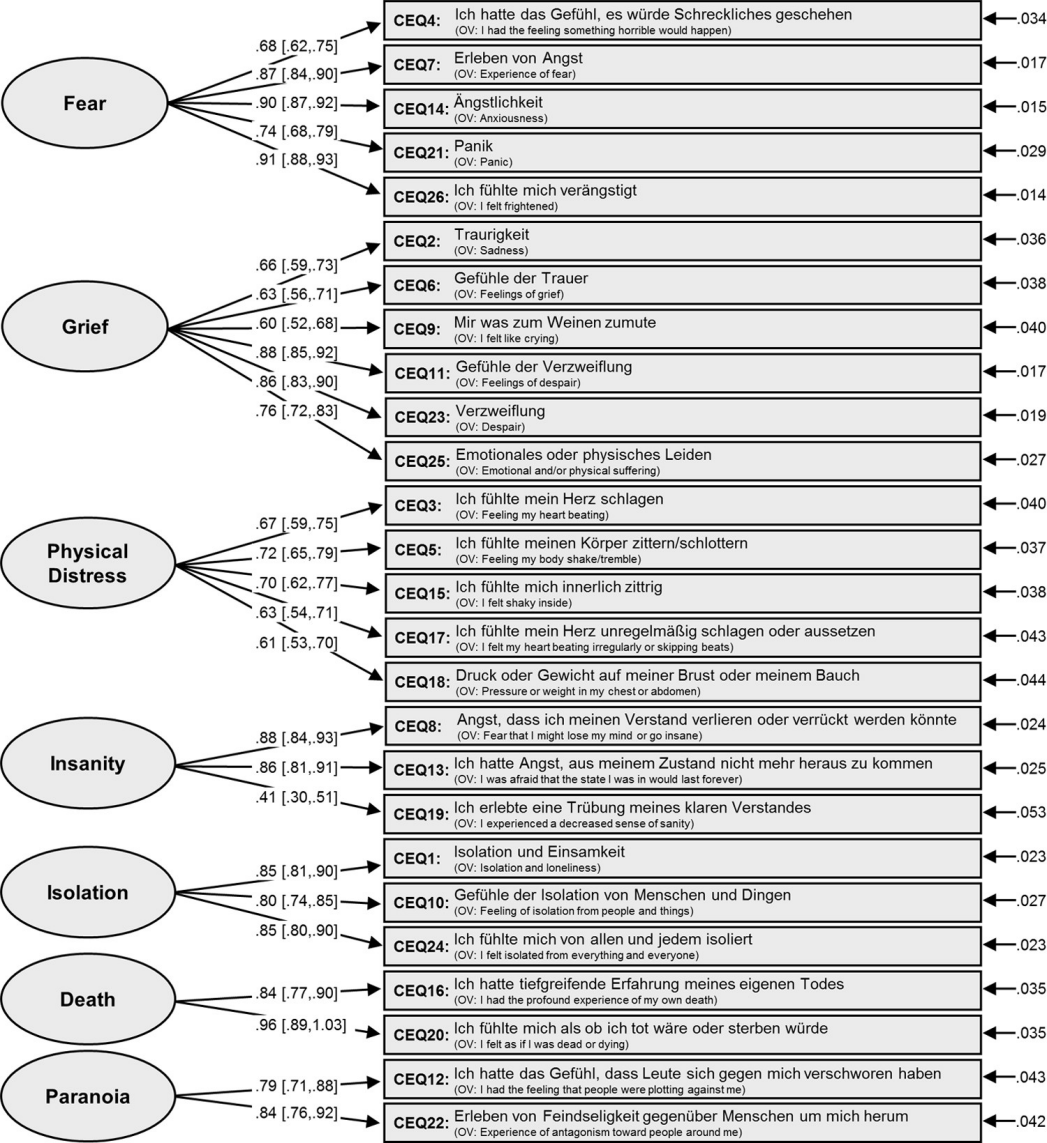
Path diagram for confirmatory factor analysis of CEQ items. This figure displays the final confirmatory factor analysis model of the CEQ with standardized loadings and error variances. Numbers in brackets are the 95% confidence intervals for the estimates.

CFA was performed to confirm and thereby validate the hypothesized factor structure underlying the German translation of the CEQ. The final CFA model with corresponding standardized factor loadings and standardized standard errors is shown in Fig 1. KMO and Bartlett’s test of sphericity indicated that the data was well suitable for factor analysis (KMO = .920, χ^2^_(288)_ = 6354.421, p < .001). To retain comparability with the original study, we employed a two-index reporting strategy [28] with cut-off values SRMR and RMSEA < .10, and CFI >. 90 [28, 29] to indicate acceptable model fit. For the present model, the following fit indices were found: SRMR = .065, RMSEA = .081 [90% confidence interval (CI) .075, .088] in the given range of acceptable model fit and the CFI = .885 very close to the suggested cut-off value, together providing initial support for internal validity.

Factorial invariance analysis was applied comprising a series of increasingly more restrictive CFAs, testing the CEQ factor structure on group variables of gender and previous struggle with a psychiatric disorder. Results show that the factor structure did not differ based on gender or prior struggle with a psychiatric disorder (Table 2) while change in fit indices for each level of factorial invariance did not exceed critical values for non-invariance, which is consistent with the findings of the original validation study. This supports strict factorial invariance and, in turn, serves as good evidence for internal validity of the CEQ factor structure.

**Table 2:** Model fit indices for factorial invariance analysis.

Model	CFI	ΔCFI	RMSEA	ΔRMSEA	SRMR	ΔSRMR
Gender						
1 (configural)	.874		.086		.074	
2 (weak)	.874	.000	.085	-.001	.076	+.002
3 (strong)	.871	-.003	.084	-.001	.076	.000
4 (strict)	.870	-.001	.084	.000	.080	+.004
Struggle with a psychiatric disorder						
1 (configural)	.873		.086		.076	
2 (weak)	.871	-.002	.085	-.001	.081	+.005
3 (strong)	.872	+.001	.084	-.001	.082	+.001
4 (strict)	.869	-.003	.084	.000	.091	+.009

CFI, comparative fit index; RMSEA, root mean square error of approximation; SRMR, standardized root mean square residual.

Critical values for the rejection of the null hypothesis of factorial invariance at any level are decrease in CFI > .010, increase in RMSEA > .015, and increase in SRMR > .010 [33]. No changes in model fit indices exceeded these values.

#### Internal consistency and convergent validity of the German CEQ

Good internal consistency was found for all CEQ subscales (Table 3). Pearson correlations between the STAI-S, the OBN (5DASC) and the CEQ total score as well as the CEQ subscale scores can be found in Table 4. Results confirmed strongly significant positive correlations between state anxiety and all but one CEQ subscale (Death), as well as the CEQ total score. The strongest positive subscale correlation of r = .59 (p < .001) was observed for CEQ subscale Fear. A correlation of r = .63 (p < .001) between the CEQ total score and STAI-S indicate a strong positive relationship between these two scales. On the other hand, correlations between the CEQ subscale scores and OBN (5DASC) mean score indicate strongly significant negative relationships between all but two CEQ subscales (Physical Distress, Death). The strongest negative correlation was observed for the factor Fear at r = -.36 (p < .001). The correlation between the CEQ total score and OBN mean score shows a highly significant negative relationship of moderate strength (r = -.31, p < .001). Comprehensively, findings provide support for good convergent validity of the German CEQ, on a subscale as well as on a global scale level.

**Table 3:** Factor correlations and factor reliabilities for the CEQ.

	Fear	Grief	Physical Distress	Insanity	Isolation	Death	Paranoia
Fear	.91						
Grief	.69(.036)	.89					
Physical Distress	.53(.052)	.47(.056)	.80				
Insanity	.65(.041)	.56(.048)	.49(.057)	.74			
Isolation	.57(.047)	.71(.037)	.31(.065)	.46(.055)	.87		
Death	.39(.055)	.34(.058)	.40(.059)	.50(.053)	.27(.061)	.89	
Paranoia	.43(.057)	.42(.059)	.47(.061)	.46(.059)	.42(.060)	.20(.066)	.80

Factor reliabilities (calculated as Cronbach’s alpha) for the entire sample are presented in italics on the diagonal at the top of the table. Correlations are presented with standard error in parentheses.

**Table 4:** Correlations (Pearson’s r) examining relationships between CEQ and the STAI-S and the OBN scale.

CEQ subscale	STAI-S		OBN	
Fear	.59	***	-.36	***
Grief	.54	***	-.25	***
Physical Distress	.42	***	*ns*	
Insanity	.43	***	-.20	***
Isolation	.48	***	-.28	***
Death	*ns*		*ns*	
Paranoia	.34	***	-.23	***
CEQ total	.63	***	-.31	***

Asterisks indicate significance level (*p < .05, **p < .01, ***p < .001); *ns*: not significant at .05.

### Results EDI

#### Sample characteristics

Sample characteristics for the total EDI validation sample are presented in Table 1. Data for participants who did not meet inclusion criteria, that is (1) reading and writing fluency in German, (2) at least 18 years of age at the time of survey completion, (3) at least one experience with a classic psychedelic, or (4) whose questionnaire ratings or free response comments at the end of the survey raised concerns about the validity of their reports were excluded from analysis. Additionally, we excluded data from participants who reported (5) to be presently struggling with a psychiatric disorder, (6) to have been struggling with more than three psychiatric disorders in the past, or (7) to have had symptoms of any type of disorder characterized by psychotic phenomenology (schizophrenia, other psychotic disorder or bipolar disorder) as we assumed potential confounding effects of such disorders with ego-dissolution. After exclusion, a final sample of 836 participants remained for the validation of the EDI who reported on 739 intense psychedelic, 151 typical psychedelic, 160 typical cocaine, and 204 typical alcohol experiences. The median time between these experiences and survey completion was 6–12 months, 6–12 months, 1–6 months, and 1–4 weeks, respectively.

#### Factor structure of the German EDI

Descriptive statistics showed that in the psychedelics subsamples two items (EII3, EII5) had a skew and kurtosis greater than 2 and 7, respectively. In the cocaine and alcohol subsample, most EDI items were nominally skewed except for EDI3 and EDI6, while statistics for the total EDI sample were below threshold. Due to significant deviation from multivariate normality (Psy-Intense: multivariate kurtosis = 71.955, z = 40.751, p < .001; Psy-Typical: multivariate kurtosis = 72.184, z = 18.479, p < .001; Cocaine: multivariate kurtosis = 203.049, z = 53.508, p < .001; Alcohol: multivariate kurtosis = 242.535, z = 72.169, p < .001), Bollen-Stine bootstrap correction (1000 samples) was applied for all subsamples. As indicated by the fit indices, the two-factor model of independent scales did not fit the data sufficiently in any of the subsamples: Psy-Intense: CFI = .809, RMSEA = .110 [90% CI .104, .116], SRMR = .107; Psy-Typical: CFI = .807, RMSEA = .123 [90% CI .109, .138], SRMR = .130; Cocaine: CFI = .813, RMSEA = .135 [90% CI .121, .149], SRMR = .137; Alcohol: CFI = .759, RMSEA = .135 [90% CI .123, .147], SRMR = .133. The hypothesized two-factor model was, therefore, rejected.

To investigate if the data suggested a multi-factorial structure or if ego-dissolution and ego-inflation items had secondary loadings onto the not intended factor, the 16 items were subjected to multiple EFAs, namely one for each of the subsamples (Psy-Intense, Psy-Typical, Cocaine, Alcohol) and one for the total sample. As EFA results were highly similar across all samples, only results for the total sample are reported in detail: KMO and Bartlett’s test of sphericity indicated that the data were well suitable for factor analysis (KMO = .885, χ^2^_(120)_ = 12382.0 p < .001). The MAP test suggested the extraction of 4 factors, while the revised MAP test, parallel analysis and inspection of the scree plot and parallel analysis suggested the extraction of 3 factors. Observed and simulated eigenvalues of the fourth component were 0.89 and 1.12, so that subsequent factor analysis extracted 3 factors, explaining 33.8%, 18.7% and 5.7% of the variance (prior to rotation).

Factor loadings for the total sample are reported in Table 5 and revealed the same pattern in the individual subsamples: Five of the EDI items had substantial loadings onto factor 2, while three items had stronger loadings onto other factors. EDI2 had the primary loading onto factor 3 and was only weakly associated with factor 2, EDI3 loaded exclusively and strongly onto the additional third factor, and EDI6 had lower loadings onto both factor 1 and 3 and was not at all associated with factor 2. With the exception of EII7 and EII8 having their primary loadings onto an additional third factor in subsample Psy-Intense and EII6 in Psy-Typical, all EII items had their highest loadings onto factor 1. Moderate to high factor loadings of ego-inflation items onto factor 1 were consistently observed across subsamples, which supported the validation of a German EII, measuring the phenomenon of ego-inflation. Factor loadings of EDI2, EDI3, and EDI6 challenged the notion of a simple structure of the German EDI, however. Loadings of items EDI1, EDI4, EDI5, EDI7, and EDI8 onto a common factor suggested the interpretation of this factor as relating to the core experience of ego-dissolution, that is, the loss of sense of self. Moderate correlation of factor 1 with factor 2 in the EFA (r = -.35) and factor 2 with factor 3 (r = .29) indicated that these factors were not independent. For all subsequent analysis, mean item scores only of items that had their primary loading onto factor 2 (in the following referred to as condensed 5-item ego-dissolution scale or EDI-5) or factor 1 (EII) were used as measures of ego-dissolution or ego-inflation, respectively. Mean item scores were strongly correlated with factor scores (regression method) for both scales: rho = .97 and .99, p < .001.

**Table 5:** Factor loadings obtained from exploratory factor analysis of 1254 responses to the German Ego-Dissolution Inventory (EDI) and additional items measuring ego-inflation (EII).

	Factor loading		
Item	2	1	3
EII1: Ich fühlte mich besonders durchsetzungsfähig	-.040	**.681**	.043
OV: I felt especially assertive			
**EDI1: Ich erlebte eine Auflösung meines „Selbst”oder Ego**	**.793**	-.019	.164
OV: I experienced a dissolution of my “self” or ego			
EII2: Ich fühlte mich wichtiger oder außergewöhnlicher als andere	.159	**.679**	-.101
OV: I felt more important or special than others			
**EDI2: Ich fühlte mich eins mit dem Universum**	**.401**	-.098	**.555**
OV: I felt at one with the universe			
EII3: Mein Ego fühlte sich aufgeblasen an	.057	**.767**	-.288
OV: My ego felt inflated			
**EDI3: Ich empfand ein Gefühl von Einigkeit mit anderen**	.158	-.045	**.660**
OV: I felt a sense of union with others			
EII4: Ich fühlte mich meiner selbst besonders sicher	-.105	**.673**	**.342**
OV: I felt especially sure-of-myself			
**EDI4: Ich erlebte meine eigene Wichtigkeit als vermindert**	**.462**	-.097	.144
OV: I experienced a decrease in my sense of self-importance			
EII5: Ich fühlte mich besonders erpicht und wetteifernd	.015	**.693**	-.208
OV: I felt especially keen and competitive			
**EDI5: Ich erlebte einen Zerfall meines „Selbst”oder Ego**	**.879**	.027	.014
OV: I experienced a disintegration of my “self” or ego			
EII6: Ich empfand meine Sicht als mehr wert als die anderer Leute	.163	**.596**	-.219
OV: I felt like my viewpoint was worth more than other peoples’			
**EDI6: Ich fühlte mich weit weniger von meinen Sorgen und Problemen vereinnahmt**	.034	**.356**	**.314**
OV: I felt far less absorbed by my own issues and concerns			
EII7: Ich fühlte mich besonders selbstbewusst	-.107	**.797**	.238
OV: I felt especially self-confident			
**EDI7: Ich verlor jegliches Ichgefühl**	**.915**	.088	-.025
OV: I lost all sense of ego			
EII8: Ich fühlte mich besonders selbstsicher	-.109	**.820**	.167
OV: I felt especially self-assured			
**EDI8: Jeglicher Begriff von Selbst und Identität zerfloss**	**.918**	.071	.041
OV: All notion of self and identity dissolved away			

Extraction method: principal axis factoring; rotation method: promax with Kaiser normalization.

Factor loadings (taken from pattern matrix) >.30 are in bold. Factor 1 was interpreted as ego-inflation, factor 2 as ego-dissolution and factor 3 did not allow for any definite qualitative interpretation.

#### Internal consistency and construct validity of the German EDI

Internal consistency of both the condensed 5-item ego-dissolution scale (EDI-5) and the ego-inflation scale (EII) proved to be good in all subsamples (Psy-Intense: alpha = .87 and .80; Psy-Typical: alpha = .87 and .82; Cocaine: alpha = .77 and .93; Alcohol: alpha = .77 and .90) and excellent in the total sample (alpha = .90 and .89). Correlations of unitive experience with ego-dissolution were of moderate strength for Psy-Intense (rho = .47 [95% CI .39, .51], p < .001) as well as for Psy-Typical (rho = .46 [95% CI .22, .56], p < .001) and were significantly different from the correlation with ego-inflation for Psy-Intense (t_(736)_ = 5.17, p < .001) and Psy-Typical (t_(148)_ = 1.98, p = .049). Convergent validity was further supported through positive correlations of 5DASC items and the PCI-derived measure of altered self-experience with ego-dissolution, which were significantly stronger than those with ego-inflation (Table 6). Unpleasant alterations in self-experience (e.g. impaired cognition) were also positively and more strongly correlated with ego-dissolution than with ego-inflation.

**Table 6:** Correlations (Spearman’s rho) examining relationships between the condensed ego-dissolution scale (EDI-5) and related ASC scales in comparison to the ego-inflation scale (EII).

	EDI-5		EII		t	p
**Psy-Intense**					df = 736	
Unitive Experience	.47	***	.23	***	5.17	< .001
Oceanic Boundlessness	.59	***	.27	***	7.99	< .001
• Experience of Unityo	.51	***	.28	***	5.39	< .001
• Disembodiment	.56	***	.12	***	10.05	< .001
Phenomenology of Consciousness	.56	***	*ns*		10.69	< .001
Dread of Ego-Dissolution	.42	***	*ns*		10.00	< .001
• Impaired Control & Cognition	.44	***	*ns*		9.98	< .001
• Anxiety	.30	***	*ns*		7.15	< .001
**Psy-Typical**					df = 148	
Unitive Experience	.46	***	.26	**	1.98	.049
Oceanic Boundlessness	.53	***	.31	***	2.36	.020
• Experience of Unity	.53	***	.27	***	2.70	.008
• Disembodiment	.47	***	*ns*		2.53	.012
Phenomenology of Consciousness	.49	***	*ns*		4.81	< .001
Dread of Ego-Dissolution	.49	***	*ns*		5.51	< .001
• Impaired Control & Cognition	.47	***	*ns*		5.48	< .001
• Anxiety	.31	***	*ns*		2.94	.004

Asterisks indicate significance level (*p < .05, **p < .01, ***p < .001); *ns*: not significant at sample-specific thresholds.

**Fig 2:**
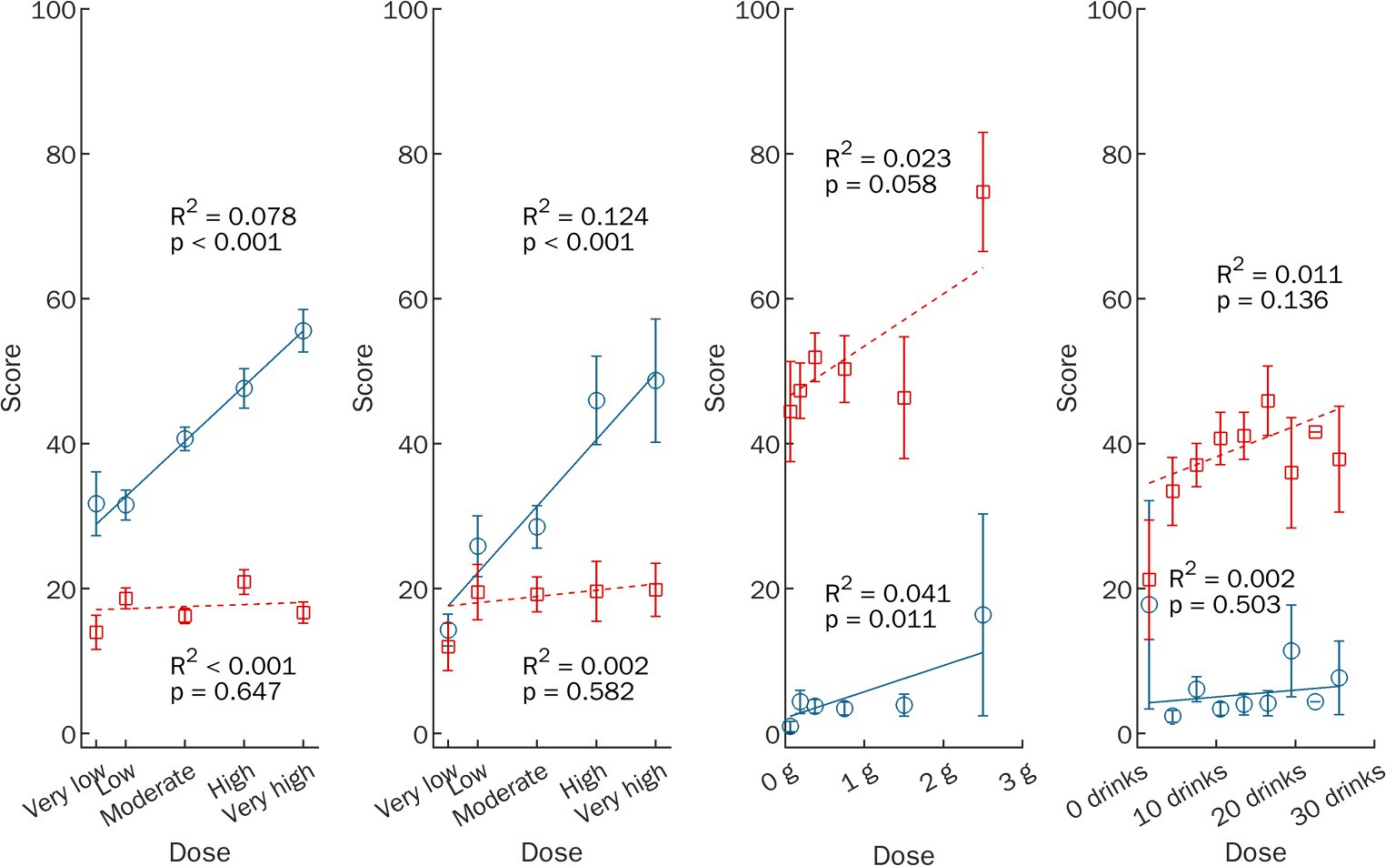
**Relationship between ego-dissolution (blue circles, solid line) or ego-inflation (red squares, dashed line), respectively, and estimated dose for different drug experiences.** The figure displays linear regression lines of best fit, the corresponding coefficient of determination and p-value as well as error bars ±1 SEM and reproduces the results reported by Nour et al. [16] for (A) most intense hallucinogen experiences (n = 678), (B) typical hallucinogen experiences (n = 137), (C) cocaine experiences (n = 160), and (D) alcohol experiences (n = 204). Data of participants who did not estimate the dose taken were excluded from analysis.

**Fig 3:**
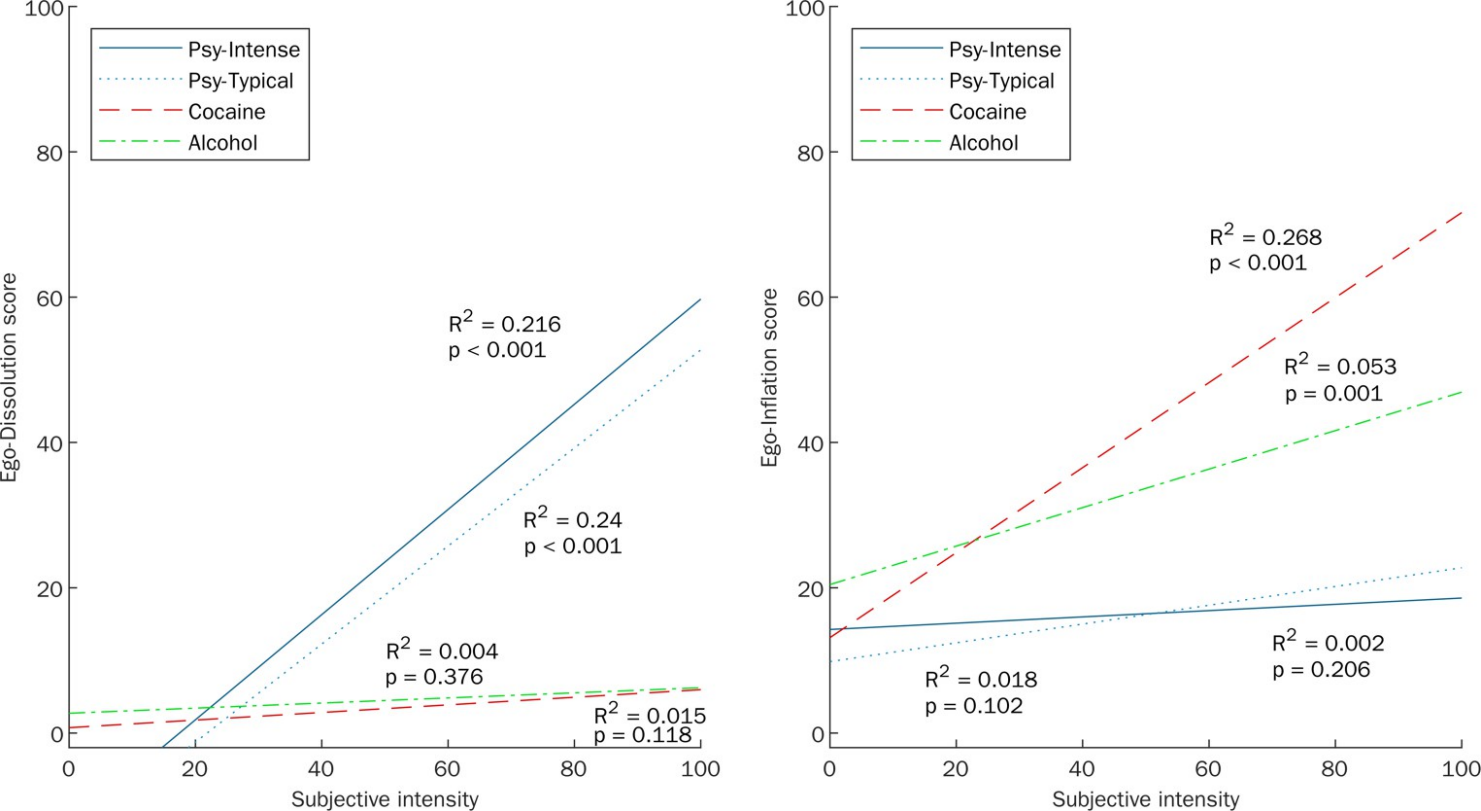
Relationship between (A) ego-dissolution or (B) ego-inflation, respectively, and subjective intensity compared across different drug experiences. This figure reproduces the results reported by Nour et al. [16] for intense hallucinogen experiences (solid blue line), typical hallucinogen experiences (light blue dotted line), cocaine experiences (red dashed line), and alcohol experiences (green dash-dot line). Intensity of the experience was rated on a visual analog scale, ranging from 0 (“not at all”) to 100 (“the most intense imaginable” for hallucinogens, “the most energized / wired imaginable” for cocaine, “the most inebriated / drunk imaginable” for alcohol).

Analysis of the relationship between ego-dissolution and psychedelics induced experiences demonstrated substantial differences compared to ego-inflation as well as compared to cocaine or alcohol induced experiences as illustrated in Figs 2 and 3: There was a significant positive correlation of both dose and intensity ratings with ego-dissolution but not with ego-inflation for Psy-Intense (rho = .27 [95% CI .21, .35] and .47 [95% CI .41, .52], both p < .001) and Psy-Typical (rho = .32 [95% CI .17, .51] and .46 [95% CI .36, .61], both p < .001). Dose and intensity did not correlate with ego-dissolution for Cocaine or Alcohol but there was a positive correlation between intensity and ego-inflation that was stronger for Cocaine (rho = .51 [95% CI .39, .61], p < .001) than for Alcohol (rho = .22 [95% CI .08, .36], p = .002). Dose-ego-dissolution and intensity-ego-dissolution correlations were significantly stronger than the dose-ego-inflation and intensity-ego-inflation correlations for both psychedelics subsamples (Psy-Intense: t_(675)_ = 4.65 and t_(736)_ = 9.80, both p < .001; Psy-Typical: t_(134)_ = 2.04, p = .044 and t_(148)_ = 3.38, p < .001). Conversely, the intensity-ego-dissolution correlation was significantly weaker than the intensity-ego-inflation correlation for Cocaine (t_(157)_ = -4.61, p < .001).

Fitting separate regression lines to compare the relationship between ego-dissolution scores (dependent variable) and ratings of subjective intensity (independent variable) across subsamples revealed that experiencing ego-dissolution was indeed predicted by intensity (F_(1,1246)_ = 227.60, MSE = 108455.6, p < .001), drug class (F_(3,1246)_ = 177.88, MSE = 84763.9, p < .001), and the interaction between intensity and drug class (F_(3,1246)_ = 33.37, MSE = 15902.9, p < .001). Slopes of the regression lines were significantly steeper for both Psy-Intense (0.724 [0.680, 0.768]) and Psy-Typical (0.675 [0.581, 0.769]) compared to Cocaine (0.052 [-0.024, 0.128]) and Alcohol (0.035 [-0.042, 0.113]; p < .001 for all comparisons) but did not differ significantly between Psy-Intense and Psy-Typical (p = .965) or Cocaine and Alcohol (p = .999), indicating a specific intensity-ego-dissolution relationship only for psychedelics induced experiences. At the same time, experiencing ego-inflation was also predicted by intensity (F_(1,1246)_ = 45.80, MSE = 16239.7, p < .001), drug class (F_(3,1246)_ = 187.60, MSE = 66520.1, p < .001), and the interaction between intensity and drug class (F_(3,1246)_ = 17.76, MSE = 6296.3, p < .001). The slope of the regression line relating ego-inflation to intensity was, however, steeper for Cocaine (0.585 [0.519, 0.651]) compared to Psy-Intense (0.043 [0.006, 0.081]) and Psy-Typical (0.129 [0.048, 0.210]; p < .001 for both comparisons) as well as Alcohol (0.265 [0.198, 0.332]; p = .004). The slopes for intense and typical psychedelic experiences did not differ significantly from each other (p = .771) but the slope for alcohol experiences was steeper compared to intense psychedelic experience (Psy-Intense: p = .020; Psy-Typical: p = .569).

**Fig 4:**
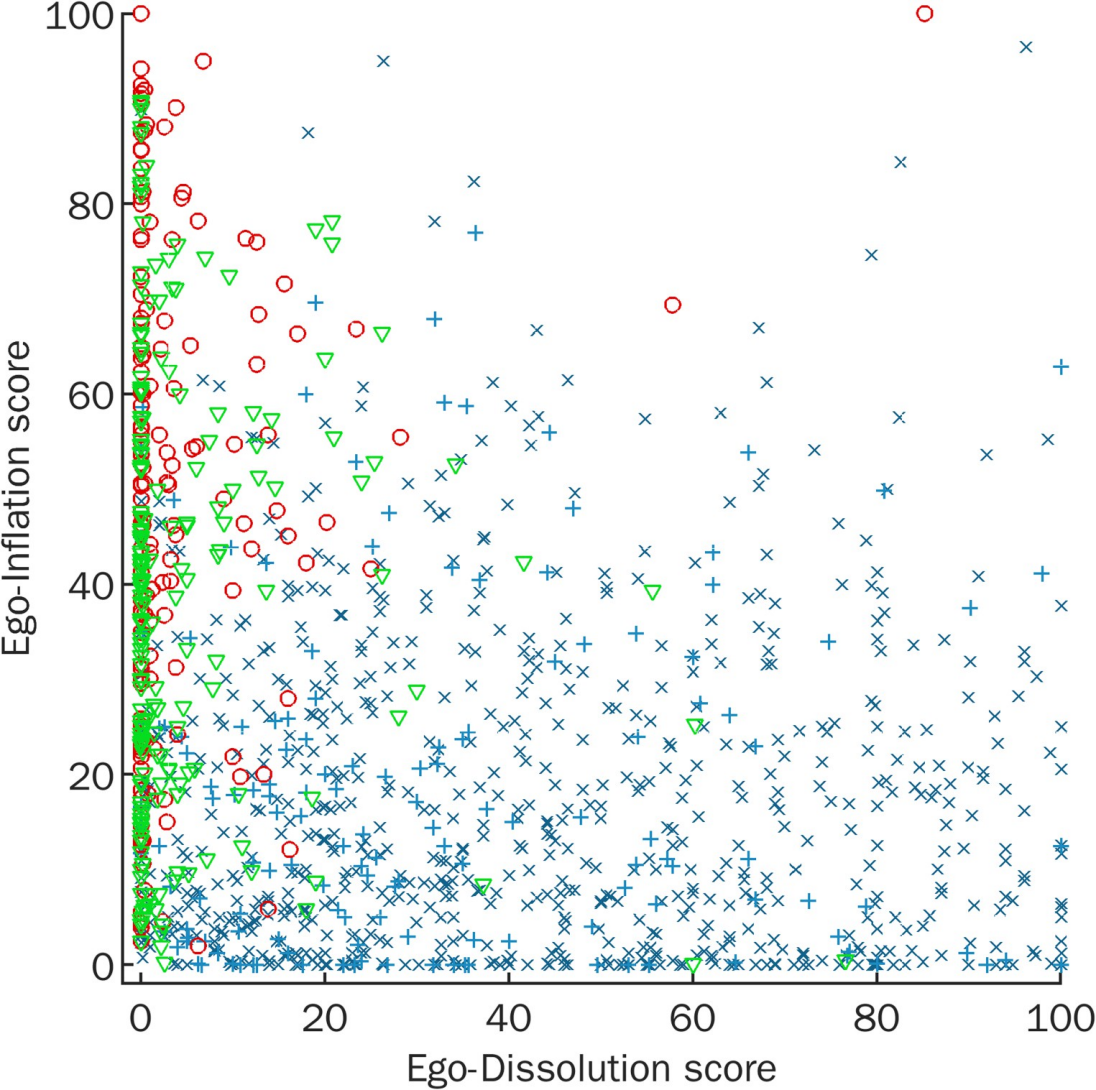
Relationship between ego-dissolution and ego-inflation for different drug experiences. Mean scores of EDI-5 and EII items were used as a measure of ego-dissolution and ego-inflation, respectively, and plotted for intense hallucinogen experiences (blue crosses), typical hallucinogen experiences (light blue plus signs), cocaine experiences (red circles), and alcohol experiences (green triangles) experiences.

Finally, the trained SVM classifier distinguished with an accuracy of 87.5% (receiver-operator characteristic (ROC) area under the curve (AUC) = .939) between typical psychedelic (n = 151) and cocaine experiences (n = 160) and with an accuracy of 85.1% (ROC AUC = .911) between typical psychedelic and alcohol experiences (n = 204). Accuracy of distinguishing between cocaine and alcohol experiences was, however, only slightly above chance level 59.6% (ROC AUC = .615). Fig 4 illustrates this relationship between ego-dissolution to ego-inflation for all drug experiences, replicating the original findings of the validation study of the English EDI.

## Discussion

In order to validate German translations of the EDI and CEQ, an online survey was conducted, in which participants retrospectively reported on their subjective experiences after the intake of psychedelics or other psychoactive drugs. Study design and data analysis were guided by the validation reports of the original English questionnaires to facilitate comparability. Results for the German CEQ demonstrated a good validation in line with the original version. Results for the German EDI could not replicate the originally proposed simple structure but supported the assumption of a condensed scale that assesses changes in self-experience specifically related to psychedelics induced experiences. Cultural difficulties in transferring concepts like ego might be a candidate explanation for our diverging results as well as difficulties in the general conceptualization of ego-dissolution.

### German version of the CEQ

In the first part of the online survey, the 26-item CEQ was used to assess challenging experiences in response to psilocybin intake. For testing the psychometric properties of the German version, the same factor structure as for the English version was assumed, comprising seven factors: (1) Fear (German: *Angst*), (2) Grief (German: *Trauer*), (3) Physical Distress (German: *Physisches Leiden*), (4) Insanity (German: *Wahnsinn*), (5) Isolation (German: *Isolation*), (6) Death (German: *Tod*), and (7) Paranoia (German: *Paranoia*). To assess internal validity, we conducted a CFA and a factorial invariance analysis; to assess internal consistency and convergent validity, we calculated Cronbach’s alpha and tested for correlations with the STAI-S, describing state anxiety, as well as OBN items of the 5DASC, describing a pleasant or blissful state.

Results of the CFA support acceptable model fit in a similar range as found for the original English validation: CFI_CEQ_ = .885 (compared to the original CFI_CEQ_en_ = .912), RMSEA_CEQ_ = .081 [90% CI .075, .088] (RMSEA_CEQ_en_ = .066 [90% CI .063, 0.070]), SRMR_CEQ_ = .065 (SRMR_CEQ_en_ = .052). Fit-indices RMSEA and SRMR fall well within the margins of the employed cut-off criteria [28] indicating good model fit. CFI was found slightly below the criterion of good fit, however still very close to the value found in the original study. Values thereby support the proposed 7-factor model. In the factorial invariance analysis, the factor structure of the German CEQ was tested in a series of increasingly more restrictive CFAs for groups of gender and previous struggle with a psychiatric disorder. Results confirmed the factor structure by demonstrating invariance; i.e. fit indices did not exceed critical values for non-invariance.

As data were collected through an anonymous online survey, the definite psychiatric health status of the participants remains inconclusive. This may potentially limit the comparability and generalizability of the study. In line with Barrett et al. [15], who demonstrated factorial invariance across these two subsamples of participants, our own results did not suggest a diverging phenomenological pattern of challenging experience for the population who endorsed having a self-reported psychiatric disorder, strengthening the finding of invariance. In summary, CFA supported an acceptable model fit and factorial invariance analysis supported good model fit. Taken together, results demonstrated internal validity. Cronbach’s alpha indicated good to excellent values of internal consistency across factors (in range alpha_CEQ_: .80–.92). Only the Insanity factor presented with a slightly lower value of alpha_CEQ_ = .73 but was still within acceptable range and close to the value from the English validation (alpha_CEQ_en_ = .76).

To assess convergent validity, we calculated Pearson correlations between CEQ subscale scores and items of the STAI-S (e.g. “I felt tense”) and items of the OBN scale of the 5DASC (e.g. “I experienced boundless pleasure”). A positive relationship between CEQ and the STAI-S items was assumed as the STAI-S items reflect experiences of anxiety, a major aspect of challenging experiences. All CEQ subscale scores (except the Death subscale) indeed showed strong and significant correlations. Predictably, the strongest correlation was found for the subscale Fear (r = .59, p < .001). Negative relationships were expected between the CEQ subscale scores and the OBN items, as OBN reflects positively experienced aspects of psychedelics induced experiences. Expectedly, all CEQ subscales except the Death subscale correlated negatively (or showed a trend towards negative correlations), with the strongest effect (r = -.36, p < .001) for Fear. Our data seem to indicate that the construct measured by the Death subscale is conceptually independent of positive and negative experiences of the psychedelics induced phenomenology. Taken together, results of the correlation analysis show strong convergent validity of the German CEQ and provide evidence for construct validity.

With regards to the variety of challenging experiences that can occur after the use of psychedelics, it is important to note that the CEQ does not necessarily cover all possible or identified facets of challenging psychedelic experiences, e.g. it does not assess aspect of confusion or cognitive impairment [47, 48]. The specific aspects of challenging experiences included in the CEQ are determined by its factor structure, which Barrett et al. [15] proposed based on two studies in the validation process. In the first study, the following categories were derived from the literature as a possible profile of challenging experiences: fear or panic, paranoia, sadness or depressed mood, anger, cognitive effects (e.g. confusion, loss of ego, loss of sanity, delusions, dissociation, depersonalization), perceptual effects (e.g. illusions), and physiological symptoms (e.g. increased heart rate, nausea/emesis, sympathetic system response). In a second study, they used EFA and selected an additional paranoia factor to narrow down and refine the selection of items based on the initial categories into the seven-factor model used in the final version of the CEQ. While the present German version confirms the same model for a standardized assessment of challenging experiences, it remains a task for future research to explore potential additional facets of challenging psychedelic experiences to potentially complete the CEQ.

### German version of the EDI

In the second part of the survey, the 8-item EDI and eight items relating to ego-inflation were used to measure changes in self-experience in response to psychedelics, cocaine, and alcohol intake. Psychometric properties were assessed in terms of factor structure, reliability, validity and the specificity of the relationship between ego-dissolution and psychedelics induced experiences. Our results provide initial evidence for the construct validity and internal consistency of a German scale measuring ego-dissolution (German: *Ich-Auflösung*). However, our factor analysis could not replicate the simple factor structure found in the original study as reported by Nour et al. [16]. CFA of data sampled with the German EDI and EII indicated a poor fit of the proposed two-factor model of two orthogonal scales that measure ego-dissolution and ego-inflation, respectively. Despite the consistently high loadings of ego-inflation items onto the first factor, subsequent EFA could not clearly separate the 16 items and suggested an alternative factor structure, where only five EDI items loaded onto the second factor, interpreted as the ego-dissolution factor, and three EDI items onto an additional third factor or the ego-inflation factor.

Using mean scores of items that were clearly associated with the second factor as a measure of ego-dissolution (EDI-5), following analysis replicated the trends of the original validation study but showed weaker correlations than the English version of the EDI: Moderate correlation between the German EDI-5 scale and the MEQ items measuring unitive experience (rho_EDI-5/MEQ-unitive_ = .46–.47, rho_EDI_en/MEQ-unitive_en_ = .74) on the one hand, and the relationship between EDI-5 and both dose (rho_EDI-5/dose_ = .27–.32, rho_EDI_en/dose_en_ = .37) and intensity (rho_EDI-5/intensity_ = .46–.47, rho_EDI_en/intensity_en_ = .58) found for psychedelics but not for cocaine or alcohol induced experiences on the other hand are consistent with the original findings and provide first evidence for construct validity. The somewhat weaker relationship between ego-dissolution and unitive experience could partly be explained by the removal of EDI2 (“I felt at one with the universe”) and EDI3 (“I felt a sense of union with others”) that are more strongly than the remaining items related to the experience of oneness. In fact, correlations between ego-dissolution when measured with the full EDI were stronger for intense as well as typical psychedelic experiences (rho_EDI-8/MEQ-unitive_ = .62–.66) in the present study. A positive relationship was also demonstrated between the EDI-5 and psychometrically validated scales, namely the 5DASC subscales Experience of Unity and Disembodiment or OBN, and selected PCI items used as a measure of altered self-experience that included the loss of sense of self (“I did not maintain a very strong sense of self-awareness at all”) and aspects of altered body perception (“I felt my body greatly expanded beyond the boundaries of my skin”). These items were selected as a blurring of the distinction between self-representation and object-representation has been discussed as being an essential aspect of the ego-dissolution experience [16]. Our results add to this notion by demonstrating that not only the unitive experience, e.g. experiencing oneself as being part of a larger whole, but also an alteration in the perception of physical boundaries, namely the own body, is closely related to ego-dissolution. On the other hand, we explored the relationship between the EDI-5 and the Impaired Control and Cognition (e.g. “I had the feeling that I no longer had a will of my own”) and Anxiety (“I experienced my surroundings as strange and weird”) or DED subscale. Items of these scales were selected as it was also discussed that alterations of ego-boundaries could be experienced as unpleasant, frightening experiences that are more similar to psychotic states [16]. We found a moderate relationship that, intriguingly, did not differ much from the relationship between ego-dissolution and unitive experience as it was only slightly weaker for intense and slightly stronger for typical psychedelic experiences (e.g. rho_EDI-5/Control&Cognition_ = .44–.47). These findings add to the demonstration of convergent validity of the EDI-5 and might also prove valuable for the conceptual framework of ego-dissolution.

Discriminant validity of the EDI-5 was supported by the specificity of the relationship of ego-dissolution for psychedelics induced experiences. This was demonstrated firstly by a significant positive correlation between ego-dissolution and dose and intensity that was found in the psychedelics subsamples but not in the cocaine or alcohol subsample; secondly by significant differences in the regression lines of intensity-ego-dissolution for psychedelics (EDI-5: 0.724–0.675, EDI_en: 0.701), cocaine (EDI-5: 0.052, EDI_en: 0.135) and alcohol (EDI-5: 0.035, EDI_en: 0.144); and thirdly by the ability of the SVM classifier to discriminate between data for psychedelics vs. cocaine and psychedelics vs. alcohol induced experiences based on EDI and EII scores with over 85% accuracy in both cases. It was shown that the EDI-5 measures alterations in the sense of self that are specifically experienced during psychedelics induced experiences and that the EII measures alterations that are specifically experienced during cocaine experiences and might to a certain degree also occur during alcohol induced experiences. Finally, excellent internal consistency was found for both the EDI-5 and the EII, suggesting a fairly similar reliability compared to the original scales (alpha_EDI-5_ = .90, alpha_EDI_en_ = .93; alpha_EII_ = .89; alpha_EII_en_ = .91).

Our results point towards preliminary validation of a German 5-item EDI, while three of the translated EDI items proved problematic. Conflicting findings in the EFA might result from sample differences, translation errors, or ambiguity in the interpretation of “ego” and “self”. Comparable with the original study, the total sample in the present study was rather homogeneous, comprising young (median = 26, IQR = 10, compared to the original study median = 28, IQR = 13), mostly male (79.1%, original: 65.6%), educated participants (28.0% had at least a bachelor’s degree, original: 61.4%) who were fairly experienced in the use of psychoactive substances. 49.5% of the present and 52.1% of the original sample had tried classic psychedelics more than 10 times in their life. Roughly half (45.9%, original: 55.7%) of the sample had taken cocaine at least twice. Data were, however, collected through an anonymous online survey rather than an experimental design, involving inaccuracies and biases resulting from self-selection, self-identified experiences, and respondents’ incomplete knowledge regarding specific substance and dose, which might have impacted comparability of the results. In the study at hand, we aimed to assess the phenomenon of ego-dissolution in a psychologically healthy population and have applied a self-report assessment of previous struggle with psychiatric disorders. Such a self-report assessment is, however, a limitation of our study as the psychiatric health status cannot be validated. On evaluating the EDI in different contexts and populations, future studies may, nevertheless, advance our knowledge about the phenomenological similarity of ego-dissolution and phenomena experienced by populations with psychotic disorders as well as the interaction between the experience of ego-dissolution and psychiatric disorders.

With regards to the finding that the EDI6 (“I felt far less absorbed by my own issues and concerns”) loaded onto the ego-inflation and the third factor, we noted that the presently applied wording of the German item (“Ich fühlte mich weit weniger von meinen Sorgen und Problemen vereinnahmt”) did not necessarily emphasize the aspect of self-reference with respect to “issues and concerns” as strongly as the wording of the original item. For future applications we therefore suggest to use the alternative wording “von meinen *eigenen* Sorgen und Problemen”. Items EDI2 (“I felt at one with the universe”) and EDI3 (“I felt a sense of union with others”) had relatively low secondary loadings onto the ego-dissolution factor, while their primary loadings were onto a third factor. Both items refer to feelings of unity and connectedness with one’s surroundings and might thereby relate more strongly to unitive experiences (as they are considered an aspect of mystical experiences) than to ego-dissolution as such. This might have been even overemphasized due to potential cultural and linguistic differences in the design of these items in their German wordings. Indeed, culturally shaped constructs such as ego-dissolution are not easily transferred to another linguistic context. A major challenge is already posed by the term “ego” and “self”, the understanding of which strongly depends on philosophical, psychological, and religious or spiritual traditions and other cultural influences. Diverging results might be a result of cross-cultural differences and require a better adaptation to the German context. Our findings could also reflect a more complex phenomenological structure of the ego-dissolution experience, potentially including multiple facets, and its relationship to unitive or mystical experiences. Including pleasant as well as adverse experiences in the conceptualization of ego-dissolution might not only help to assess a substance’s ego-dissolving or ego-inflating potential as suggested by Nour et al. [16] but also enable assessments under which circumstances (dose, set, setting) an ego-dissolving substance will realize its beneficial therapeutic effects (rather resembling a mystical experience) and under which circumstances it will not (rather resembling psychotic states). Further studies are, however, needed to refine the conceptual and operational definition of a German measure of ego-dissolution.

## Conclusions

In sum, the translated German version of the CEQ demonstrated a stable factor structure in line with the original English version. We were able to show indicators for good reliability as well as validity, which encourages the application of the German CEQ in future studies. With regards to the German version of the EDI, we did not find the factor structure of the English version confirmed. Five items of the German version of the EDI demonstrated good validity and reliability and can be used as a unidimensional measure of ego-dissolution. The German translation of eight ego-inflation items (EII) was found to be a single-factor measure of increased self-assuredness with excellent reliability. To compare and further investigate its psychometric structure, we recommend the use of the full 8-item EDI with the suggested change for EDI6 in future studies.

## Supporting information

S1 Appendix Challenging Experience Questionnaire. (PDF)

S2 Appendix Ego-Dissolution Inventory. (PDF)

S3 Appendix List of survey items. (PDF)

S1 Dataset CEQ data. (ZIP)

S2 Dataset EDI data. (ZIP)
