# Supplementary material for: Phenomenological assessment of psychedelics induced experiences: Translation and validation of the German Challenging Experience Questionnaire (CEQ) and Ego-Dissolution Inventory (EDI)
Source: PLoS One. 2022 Mar 16;17(3):e0264927. doi: 10.1371/journal.pone.0264927 (PMC8926265; doi:10.1371/journal.pone.0264927)
Supplement: S3 Appendix — (PDF) [file pone.0264927.s003.pdf]

S3 Appendix. List of survey items

The survey consisted of two parts. After providing general demographic information and information on prior substance use, participants reported on (1) psilocybin induced challenging experiences for the validation of the CEQ and / or (2a) intense psychedelic induced experiences (2b), typical psychedelic induced experiences (2b), typical cocaine experiences (2c) and / or typical alcohol induced experiences (2d) for the validation of the EDI.

Table A. Items included in our online survey Study on Phenomena of Hallucinogen Induced Experiences (SPHINX)

**Demographic information and substance use**		
Question	Response options	
Bitte geben Sie Ihre Alter an.	Alter in Jahren ___	age
Bitte geben Sie Ihr Geschlecht an.	Männlich<br>Weiblich<br>Anderes (bitte angeben) ___	gender
Bitte geben Sie Ihren höchsten Bildungsabschluss an.	Ohne Schulabschluss<br>Hauptschulabschluss<br>Mittlerer Schulabschluss (z.B. Realschulabschluss)<br>Fachhochschulreife (Abschluss einer Fachoberschule)<br>Allgemeine Hochschulreife (Abitur)<br>Abschluss einer Kunst- oder Musikhochschule<br>Hochschulabschluss (Diplom)<br>Hochschulabschluss (Bachelor)<br>Hochschulabschluss (Magister)<br>Hochschulabschluss (Master)<br>Hochschulabschluss (Promotion)<br>Hochschulabschluss (Habilitation)	education
Bitte geben Sie Ihren Familienstand an.	Alleinstehend<br>In einer festen Beziehung	relationship
Wie hoch ist Ihr durchschnittliches Haushaltsnettoeinkommen im Monat?	<900€<br>900-1299€<br>1300-1499€<br>1500-1999€<br>2000-2599€<br>2600-3599€<br>3600-4999€<br>5000-7499€<br>7500-18000€<br>>18000€	income
Bitte geben Sie Ihr ungefähres Körpergewicht an.	Gewicht in kg ___	weight
Wie viele Zigaretten rauchen Sie durchschnittlich pro Tag?	Zigaretten pro Tag ___	cigs_day
An wie vielen Tagen pro Woche nehmen Sie gewöhnlich Alkohol zu sich?	Tage pro Woche ___	alc_day
Wie viele alkoholische Getränke nehmen Sie pro Woche zu sich?<br><br>1 Getränk entspricht z.B. 0,125l Wein oder 0,33l Bier oder 4cl Spirituosen	Getränke pro Woche ___	drinks_week

Leiden Sie gegenwärtig unter einer psychischen Erkrankung?	Nein<br>Ja, und zwar ____	disorder_present
Bitte wählen Sie alle Bitte wählen Sie alle psychischen Erkrankungen aus, unter denen Sie in der Vergangenheit gelitten haben.	Keine<br>Angststörung (Phobien, Panikstörung, generalisierte Angststörung)<br>Posttraumatische Belastungsstörung (PTSD)<br>Depressionen<br>Bipolare Störung<br>Alkoholabhängigkeit<br>Substanzmissbrauch (nicht Alkoholabhängigkeit)<br>Essstörung<br>Zwangsstörung<br>Schizophrenie<br>Aufmerksamkeitsdefizit-/Hyperaktivitätsstörung (ADHS)<br>Psychotische Störung	disorder_past
Wie oft haben Sie die folgenden Substanzen in Ihrem ganzen Leben zu sich genommen?	• Cannabis (Hanf, Marihuana, Haschisch)<br>• Ecstasy, MDMA, MDE, MDA o.Ä.<br>• LSD<br>• Psilocybin, psilocybinhaltige Pilze<br>• Amphetamin und Methamphetamin (Speed, Ice, Crystal, ähnliche Stimulantien)<br>• Kokain<br>• Heroin, andere Opiate<br>• Peyote, Meskalin<br>• DMT, Ayahuasca<br>• Schnüffelstoffe, Poppers<br>• Medikamente (z.B. Schlafmittel, Schmerzmittel, Ritalin) wegen psychoaktiver Wirkung<br>• Andere Substanzen (z.B. Ketamin, Crack, GHB, Lachgas, PCP, Stechapfel, Bilsenkraut, Tollkirsche)<br><br>Nie; Nur 1 Mal; 2-5 Mal; 6-10 Mal; 11-15 Mal; 16-25 Mal; 26-50 Mal; 51-100 Mal; 101-1000 Mal; >1000 Mal; k. A	use_lifetime
Wie oft haben Sie die folgenden Substanzen im letzten halben Jahr zu sich genommen?	• Cannabis (Hanf, Marihuana, Haschisch)<br>• Ecstasy, MDMA, MDE, MDA o.Ä.<br>• LSD<br>• Psilocybin, psilocybinhaltige Pilze<br>• Amphetamin und Methamphetamin (Speed, Ice, Crystal, ähnliche Stimulantien)<br>• Kokain<br>• Heroin, andere Opiate<br>• Peyote, Meskalin<br>• DMT, Ayahuasca<br>• Schnüffelstoffe, Poppers<br>• Medikamente (z.B. Schlafmittel, Schmerzmittel, Ritalin) wegen psychoaktiver Wirkung<br>• Andere Substanzen (z.B. Ketamin, Crack, GHB, Lachgas, PCP, Stechapfel, Bilsenkraut, Tollkirsche)<br><br>Nie; 1-2 Mal/halbes Jahr; 3-5 Mal/halbes Jahr; 1-3 Mal/Monat; 1-2/Woche; 3-6/Woche; Täglich; >1Mal/Tag; k.A	use_past_six_months

Psilocybin induced challenging experiences for validation of the CEQ		
Haben Sie nach der Einnahme von Psilocybin oder psilocybinhaltigen Pilzen / Magic Mushrooms / Zauberpilzen jemals ein psychisch belastendes oder herausforderndes Erlebnis erfahren?<br>Hatten Sie z.B. einen Bad Trip/Horrortrip?	Ja, ich hatte ein herausforderndes Erlebnis nach der Einnahme von Psilocybin.<br>Nein, ich hatte noch kein herausforderndes Erlebnis nach der Einnahme von Psilocybin, aber nach der Einnahme eines anderen Halluzinogens.<br>Ich hatte noch nie ein herausforderndes Erlebnis nach der Einnahme von Psilocybin oder eines anderen Halluzinogens.	ce_filter
Wie alt waren Sie, als Sie das erste Mal Psilocybin [Oder: dieses Halluzinogen] eingenommen haben?	Alter in Jahren ___	first_psilo
Wie alt waren Sie, als Sie das letzte Mal Psilocybin [Oder: dieses Halluzinogen] eingenommen haben?	Alter in Jahren ___	last_psilo
Wie alt waren Sie, als Sie das herausfordernde Erlebnis hatten?	Alter in Jahren ___	age_ce
Bitte geben Sie die eingenommene Psilocybindosis an. Nennen Sie die Darreichungsform und schätzen Sie die Menge, z.B. das Gewicht getrockneter Pilze.<br>[Oder: Bitte nennen Sie das Halluzinogen, das Sie eingenommen haben, und schätzen Sie die Dosis.]	Darreichungsform ___<br>Menge ___<br><br>[Oder: Halluzinogen ___<br>Dosis ___]	dose
Isolation und Einsamkeit	0 - kein(e), gar nicht<br>1 - so schwach, kann ich nicht genau sagen<br>2 - schwach<br>3 - mittel<br>4 - stark<br>5 - extrem (mehr als jemals zuvor in meinem Leben)	ceq1
Traurigkeit	0 - kein(e), gar nicht<br>1 - so schwach, kann ich nicht genau sagen<br>2 - schwach<br>3 - mittel<br>4 - stark<br>5 - extrem (mehr als jemals zuvor in meinem Leben)	ceq2
Ich fühlte mein Herz schlagen	0 - kein(e), gar nicht<br>1 - so schwach, kann ich nicht genau sagen<br>2 - schwach<br>3 - mittel<br>4 - stark<br>5 - extrem (mehr als jemals zuvor in meinem Leben)	ceq3
Ich hatte das Gefühl, es würde Schreckliches geschehen	0 - kein(e), gar nicht<br>1 - so schwach, kann ich nicht genau sagen<br>2 - schwach<br>3 - mittel<br>4 - stark<br>5 - extrem (mehr als jemals zuvor in meinem Leben)	ceq4
Ich fühlte meinen Körper zittern/schlottern.	0 - kein(e), gar nicht<br>1 - so schwach, kann ich nicht genau sagen<br>2 - schwach<br>3 - mittel<br>4 - stark<br>5 - extrem (mehr als jemals zuvor in meinem Leben)	ceq5

Gefühle der Trauer	0 - kein(e), gar nicht<br>1 - so schwach, kann ich nicht genau sagen<br>2 - schwach<br>3 - mittel<br>4 - stark<br>5 - extrem (mehr als jemals zuvor in meinem Leben)	ceq6
Erleben von Angst	0 - kein(e), gar nicht<br>1 - so schwach, kann ich nicht genau sagen<br>2 - schwach<br>3 - mittel<br>4 - stark<br>5 - extrem (mehr als jemals zuvor in meinem Leben)	ceq7
Angst, dass ich meinen Verstand verlieren oder verrückt werden könnte	0 - kein(e), gar nicht<br>1 - so schwach, kann ich nicht genau sagen<br>2 - schwach<br>3 - mittel<br>4 - stark<br>5 - extrem (mehr als jemals zuvor in meinem Leben)	ceq8
Mir war zum Weinen zumute	0 - kein(e), gar nicht<br>1 - so schwach, kann ich nicht genau sagen<br>2 - schwach<br>3 - mittel<br>4 - stark<br>5 - extrem (mehr als jemals zuvor in meinem Leben)	ceq9
Gefühl der Isolation von Menschen und Dingen	0 - kein(e), gar nicht<br>1 - so schwach, kann ich nicht genau sagen<br>2 - schwach<br>3 - mittel<br>4 - stark<br>5 - extrem (mehr als jemals zuvor in meinem Leben)	ceq10
Gefühle der Verzweiflung	0 - kein(e), gar nicht<br>1 - so schwach, kann ich nicht genau sagen<br>2 - schwach<br>3 - mittel<br>4 - stark<br>5 - extrem (mehr als jemals zuvor in meinem Leben)	ceq11
Ich hatte das Gefühl, dass Leute sich gegen mich verschworen haben	0 - kein(e), gar nicht<br>1 - so schwach, kann ich nicht genau sagen<br>2 - schwach<br>3 - mittel<br>4 - stark<br>5 - extrem (mehr als jemals zuvor in meinem Leben)	ceq12
Ich hatte Angst, aus meinem Zustand nicht mehr herauszukommen	0 - kein(e), gar nicht<br>1 - so schwach, kann ich nicht genau sagen<br>2 - schwach<br>3 - mittel<br>4 - stark<br>5 - extrem (mehr als jemals zuvor in meinem Leben)	ceq13
Ängstlichkeit	0 - kein(e), gar nicht<br>1 - so schwach, kann ich nicht genau sagen<br>2 - schwach<br>3 - mittel<br>4 - stark<br>5 - extrem (mehr als jemals zuvor in meinem Leben)	ceq14
Ich fühlte mich innerlich zittrig	0 - kein(e), gar nicht<br>1 - so schwach, kann ich nicht genau sagen<br>2 - schwach<br>3 - mittel<br>4 - stark<br>5 - extrem (mehr als jemals zuvor in meinem Leben)	ceq15

Ich hatte die tiefgreifende Erfahrung meines eigenen Todes	0 - kein(e), gar nicht<br>1 - so schwach, kann ich nicht genau sagen<br>2 - schwach<br>3 - mittel<br>4 - stark<br>5 - extrem (mehr als jemals zuvor in meinem Leben)	ceq16
Ich fühlte mein Herz unregelmäßig schlagen oder aussetzen	0 - kein(e), gar nicht<br>1 - so schwach, kann ich nicht genau sagen<br>2 - schwach<br>3 - mittel<br>4 - stark<br>5 - extrem (mehr als jemals zuvor in meinem Leben)	ceq17
Druck oder Gewicht auf meiner Brust oder meinem Bauch	0 - kein(e), gar nicht<br>1 - so schwach, kann ich nicht genau sagen<br>2 - schwach<br>3 - mittel<br>4 - stark<br>5 - extrem (mehr als jemals zuvor in meinem Leben)	ceq18
Ich erlebte eine Trübung des klaren Verstandes	0 - kein(e), gar nicht<br>1 - so schwach, kann ich nicht genau sagen<br>2 - schwach<br>3 - mittel<br>4 - stark<br>5 - extrem (mehr als jemals zuvor in meinem Leben)	ceq19
Ich fühlte mich, als ob ich tot wäre oder sterben würde	0 - kein(e), gar nicht<br>1 - so schwach, kann ich nicht genau sagen<br>2 - schwach<br>3 - mittel<br>4 - stark<br>5 - extrem (mehr als jemals zuvor in meinem Leben)	ceq20
Panik	0 - kein(e), gar nicht<br>1 - so schwach, kann ich nicht genau sagen<br>2 - schwach<br>3 - mittel<br>4 - stark<br>5 - extrem (mehr als jemals zuvor in meinem Leben)	ceq21
Erleben von Feindseligkeit gegenüber Menschen um mich herum	0 - kein(e), gar nicht<br>1 - so schwach, kann ich nicht genau sagen<br>2 - schwach<br>3 - mittel<br>4 - stark<br>5 - extrem (mehr als jemals zuvor in meinem Leben)	ceq22
Verzweiflung	0 - kein(e), gar nicht<br>1 - so schwach, kann ich nicht genau sagen<br>2 - schwach<br>3 - mittel<br>4 - stark<br>5 - extrem (mehr als jemals zuvor in meinem Leben)	ceq23
Ich fühlte mich von allem und jedem isoliert	0 - kein(e), gar nicht<br>1 - so schwach, kann ich nicht genau sagen<br>2 - schwach<br>3 - mittel<br>4 - stark<br>5 - extrem (mehr als jemals zuvor in meinem Leben)	ceq24
Emotionales und / oder physisches Leiden	0 - kein(e), gar nicht<br>1 - so schwach, kann ich nicht genau sagen<br>2 - schwach<br>3 - mittel<br>4 - stark<br>5 - extrem (mehr als jemals zuvor in meinem Leben)	ceq25

Ich fühlte mich verängstigt	0 - kein(e), gar nicht<br>1 - so schwach, kann ich nicht genau sagen<br>2 - schwach<br>3 - mittel<br>4 - stark<br>5 - extrem (mehr als jemals zuvor in meinem Leben)	ceq26
Ich hatte das Gefühl, dass über mich geredet wurde.	0 - kein(e), gar nicht<br>1 - so schwach, kann ich nicht genau sagen<br>2 - schwach<br>3 - mittel<br>4 - stark<br>5 - extrem (mehr als jemals zuvor in meinem Leben)	psi7
Mir schien, dass Leute Dinge sagten und taten, um mich zu verärgern.	0 - kein(e), gar nicht<br>1 - so schwach, kann ich nicht genau sagen<br>2 - schwach<br>3 - mittel<br>4 - stark<br>5 - extrem (mehr als jemals zuvor in meinem Leben)	psi11
Ich war von dem Gedanken beunruhigt, dass Leute mich beobachteten.	0 - kein(e), gar nicht<br>1 - so schwach, kann ich nicht genau sagen<br>2 - schwach<br>3 - mittel<br>4 - stark<br>5 - extrem (mehr als jemals zuvor in meinem Leben)	psi14
Ich fühlte mich, als hätten Leute es auf mich abgesehen.	0 - kein(e), gar nicht<br>1 - so schwach, kann ich nicht genau sagen<br>2 - schwach<br>3 - mittel<br>4 - stark<br>5 - extrem (mehr als jemals zuvor in meinem Leben)	psi17
Ich hatte ein vages Gefühl der Bedrohung oder ein plötzliches Angstgefühl aus Gründen, die ich nicht verstand.	0 - kein(e), gar nicht<br>1 - so schwach, kann ich nicht genau sagen<br>2 - schwach<br>3 - mittel<br>4 - stark<br>5 - extrem (mehr als jemals zuvor in meinem Leben)	psi38
Ich hatte das Gefühl, dass Leute hinter meinem Rücken über mich lachten.	0 - kein(e), gar nicht<br>1 - so schwach, kann ich nicht genau sagen<br>2 - schwach<br>3 - mittel<br>4 - stark<br>5 - extrem (mehr als jemals zuvor in meinem Leben)	psi42
Ich war verkrampft.	Überhaupt nicht<br>Ein wenig<br>Ziemlich<br>Sehr	stai14
Ich fühlte mich geborgen.	Überhaupt nicht<br>Ein wenig<br>Ziemlich<br>Sehr	stai2
Ich fühlte mich wohl.	Überhaupt nicht<br>Ein wenig<br>Ziemlich<br>Sehr	stai10
Ich war überreizt.	Überhaupt nicht<br>Ein wenig<br>Ziemlich<br>Sehr	stai18

Ich fühlte mich angespannt.	Überhaupt nicht<br>Ein wenig<br>Ziemlich<br>Sehr	stai3
Ich empfand eine allumfassende Liebe.	Nein, nicht mehr als gewöhnlich — Ja, ich erlebte dies komplett/vollständig	5dasc91
Ich empfand tiefen Frieden in mir.	Nein, nicht mehr als gewöhnlich — Ja, ich erlebte dies komplett/vollständig	5dasc86
Ich empfand grenzenlose Freude.	Nein, nicht mehr als gewöhnlich — Ja, ich erlebte dies komplett/vollständig	5dasc12
Körperliche Empfindungen waren sehr lustvoll.	Nein, nicht mehr als gewöhnlich — Ja, ich erlebte dies komplett/vollständig	5dasc3
Wie stark psychisch belastend oder herausfordernd war das Erlebnis?	Nicht mehr als gewöhnliche, alltägliche Erlebnisse<br>Vergleichbar mit belastenden oder herausfordernden Erlebnissen, die sich durchschnittlich einmal pro Woche ereignen<br>Vergleichbar mit belastenden oder herausfordernden Erlebnissen, die sich durchschnittlich einmal pro Monat ereignen<br>Vergleichbar mit belastenden oder herausfordernden Erlebnissen, die sich durchschnittlich einmal pro Jahr ereignen<br>Vergleichbar mit belastenden oder herausfordernden Erlebnissen, die sich durchschnittlich einmal alle 5 Jahre ereignen<br>Unter den 10 belastendsten oder herausforderndsten Erlebnissen meines Lebens<br>Unter den 5 belastendsten oder herausforderndsten Erlebnissen meines Lebens<br>Das belastendste oder herausforderndste Erlebnis meines Lebens	how_challenging
Wie bedeutungsvoll war das Erlebnis für Sie persönlich?	Nicht mehr als gewöhnliche, alltägliche Erlebnisse<br>Vergleichbar mit bedeutungsvollen Erlebnissen, die sich durchschnittlich einmal pro Woche ereignen<br>Vergleichbar mit bedeutungsvollen Erlebnissen, die sich durchschnittlich einmal pro Monat ereignen<br>Vergleichbar mit bedeutungsvollen Erlebnissen, die sich durchschnittlich einmal pro Jahr ereignen<br>Vergleichbar mit bedeutungsvollen Erlebnissen, die sich durchschnittlich einmal alle 5 Jahre ereignen<br>Unter den 10 bedeutungsvollsten Erlebnissen meines Lebens<br>Unter den 5 bedeutungsvollsten Erlebnissen meines Lebens<br>Das bedeutungsvollste Erlebnis meines Lebens	how_meaningful
Bitte geben Sie an, inwieweit das Erlebnis für Sie spirituell bedeutsam war.	Kann ich nicht sagen<br>Gar nicht<br>Schwach<br>Mittel<br>Stark<br>Unter den 5 spirituell bedeutsamsten Erlebnissen meines Lebens<br>Das spirituell bedeutsamste Erlebnis meines Lebens	how_spiritual

Glauben Sie, dass das Erlebnis und die Verarbeitung des Erlebnisses eine Veränderung Ihres derzeitigen persönlichen Wohlbefindens oder Ihrer Lebenszufriedenheit bewirkt hat?	Sehr stark vermindert<br>Mäßig vermindert<br>Schwach vermindert<br>Keine Veränderung<br>Schwach gesteigert<br>Mäßig gesteigert<br>Sehr stark gesteigert	change_wellbeing
Würden Sie diese Erfahrung einschließlich des herausfordernden Erlebnisses wiederholen?	Ja<br>Nein	repeat
Bitte markieren Sie alle Halluzinogene, mit denen Sie vor dem herausfordernden Erlebnis bereits Erfahrungen gemacht hatten.	Keine vorherigen Erfahrungen<br>LSD<br>Psilocybin, psilocybinhaltige Pilze<br>DMT, Ayahuasca<br>Meskalin, Peyote<br>Sonstiges (bitte angeben) ___	prior_experience
Haben Sie in den Wochen vor dem herausfordernden Erlebnis unter Depressionen oder depressiven Verstimmungen gelitten?	Ja<br>Nein	struggle_depression
Haben Sie in den Wochen vor dem herausfordernden Erlebnis unter Angstzuständen gelitten?	Ja<br>Nein	struggle_anxiety
In welcher Absicht haben Sie das Halluzinogen eingenommen?	Ich habe das Halluzinogen nur eingenommen, weil andere Leute das Halluzinogen einnahmen, ich empfand eine Art Gruppenzwang, hatte aber keine ernsthafte Absicht;<br>Neugier ohne irgendeine andere ernsthafte Absicht;<br>Freizeitvergnügen, z.B. um das Erlebnis zu genießen, welches nicht unbedingt aber auch ein soziales Event wie ein Konzert einschließt;<br>Eine ernsthafte Absicht zur psychologischen Selbsterforschung, z.B. um ein persönliches Problem zu lösen oder Selbstverständigung zu steigern;<br>Eine ernsthafte Absicht zur Erkundung von Spiritualität oder des Heiligen, z.B. um Gott oder eine höhere Macht oder das Wesen der letzten Wirklichkeit zu beschauen;<br>Sonstiges (bitte angeben) ___	intention
Haben Sie unmittelbar vor oder während des herausfordernden Erlebnisses Cannabis konsumiert?	Nein<br>Ja	use_cannabis
Haben Sie unmittelbar vor oder während des herausfordernden Erlebnisses eine andere Substanz konsumiert?	Nein<br>Ja und zwar (bitte angeben) ___	use_other

Bitte bewerten Sie den sozialen Support während des herausfordernden Erlebnisses.	Sehr stark negativ<br>Mäßig negativ<br>Schwach negativ<br>Keine Auswirkung, neutral<br>Schwach positiv<br>Mäßig positiv<br>Sehr stark positiv	social_support
Bitte beschreiben Sie kurz die Art des sozialen Supports.<br>War ein Tripsitter anwesend?<br>Waren die anwesenden Personen nüchtern?<br>Hatten die anwesenden Personen ebenfalls ein Halluzinogen oder eine andere Substanz eingenommen? Wenn ja, welche(s)?	Freitextfeld	social_support_comment
In welcher Umgebung hatten Sie das herausfordernde Erlebnis?	Bei mir zuhause<br>Bei einem*r Freund*in oder Bekannten zuhause<br>Auf einer privaten Party<br>Auf einer öffentlichen Party oder einem Festival<br>In der Natur<br>Sonstiges (bitte angeben) ___	environment
Bitte bewerten Sie, inwieweit sich die Umgebung auf Ihr körperliches Wohlbefinden ausgewirkt hat.	Sehr stark negativ<br>Mäßig negativ<br>Schwach negativ<br>Keine Auswirkung, neutrale Umgebung<br>Schwach positiv<br>Mäßig positiv<br>Sehr stark positiv	environment_impact
Wie viele Personen waren während des herausfordernden Erlebnisses anwesend?	Ich war allein<br>1-3<br>4-8<br>9-50<br>>50	others_present
Wie würden Sie die Beziehung zu den anwesenden Personen am ehesten beschreiben?	Ich war allein<br>Unbekannte<br>Bekannte<br>Freunde<br>Enge Vertraute oder Partner:in<br>Sonstiges (bitte angeben) ___	others_relationship
Wie lange dauerte das herausfordernde Erlebnis an?	Während der gesamten Wirkungsdauer des Halluzinogens<br>>2 Stunden, aber nicht während der gesamten Wirkungsdauer des Halluzinogens<br>1-2 Stunden<br>30-60 Minuten<br>10-30 Minuten<br><10 Minuten<br>k.A.	ce_duration

Bitte markieren Sie alle Maßnahmen, die Sie ergriffen haben, um das herausfordernde Erlebnis zu mildern.	Keine<br>Versuch, mich mental zu beruhigen<br>Ortswechsel<br>Bewegung oder Körperkontakt zu anderen<br>Musikwechsel<br>Wechsel des sozialen Umfelds<br>Freund*in um Hilfe gebeten<br>Eingriff eines Guides/Tripsitters<br>Cannabiskonsum<br>Alkoholkonsum<br>Einnahme einer anderen Substanz Musikwechsel<br>Sonstiges (bitte angeben) ___	measures
Bitte markieren Sie alle ergriffenen Maßnahmen, die erfolgreich waren.	Keine<br>Versuch, mich mental zu beruhigen<br>Ortswechsel<br>Bewegung oder Körperkontakt zu anderen<br>Musikwechsel<br>Wechsel des sozialen Umfelds<br>Freund*in um Hilfe gebeten<br>Eingriff eines Guides/Tripsitters<br>Cannabiskonsum<br>Alkoholkonsum<br>Einnahme einer anderen Substanz Musikwechsel<br>Sonstiges (bitte angeben) ___	measures_successful
Welche akuten Auswirkungen hatte das herausfordernde Erlebnis?	Keine<br>Ich habe andere oder mich selbst der Gefahr ausgesetzt, physischen Schaden zu nehmen.<br>Ich verhielt mich anderen oder mir selbst gegenüber in einer physisch aggressiven oder gewalttätigen Weise.<br>Ich verhielt mich anderen oder mir selbst gegenüber in einer verbal aggressiven oder feindseligen Weise.<br>Ich musste eine Notaufnahme oder ein Krankenhaus aufsuchen.<br>Sonstiges (bitte angeben) ___	acute_symptoms
Welche der folgenden psychischen oder emotionalen Symptome traten nachträglich auf?	Keine<br>Furcht<br>Angst<br>Sonstiges (bitte angeben)<br>Depressionen<br>Paranoia<br>Psychose	post_symptoms
Falls zutreffend: Wie lange dauerten diese Symptome an?	Furcht ___<br>Angst ___<br>Depressionen ___<br>Paranoia ___<br>Psychose ___<br>Sonstige ___	post_symptoms_duration
Falls zutreffend: Wurde für eines dieser Symptome (Furcht, Angst, Depressionen, Paranoia, Psychose, sonstige) eine professionelle Behandlung notwendig?	Ja<br>Nein	intervention

**Psychedelic (P), cocaine (C) and alcohol (A) induced experiences for validation of the EDI**		
Wie lange liegt das Erlebnis zurück?	<24 Stunden<br>1-7 Tage<br>1-4 Wochen<br>1-6 Monate<br>6-12 Monate<br>1-5 Jahre<br>6-10 Jahre<br>>10 Jahre	time_elapsed
Bitte wählen Sie das eingenommene Halluzinogen und schätzen Sie die Dosis, z. B. LSD: ein halbes Ticket/ca. 50 Mikrogramm.[P]	• LSD<br>• Psilocybin, psilocybinhaltige Pilze<br>• DMT, Ayahuasca<br>• Meskalin, Peyote<br><br>Darreichungsform & geschätzte Dosis ___	psychedelic + dose
Bitte schätzen Sie die eingenommene Dosis.[C]	< 1/8 g (ungefähr 1 mittlere Line oder 2 kleinere Lines)<br>1/8-1/4 g (ungefähr 2 mittlere Lines oder 1 größere Line)<br>1/4-1/2 g (ungefähr 4 mittlere Lines oder 2 größere Lines)<br>1/2-1 g (bis zu 10 mittlere Lines oder 4-5 größere Lines)<br>1-2 g (bis zu 20 mittlere Lines oder 8-10 größere Lines)<br>> 2 g	dose
Bitte schätzen Sie, wie viele alkoholische Getränke Sie zu sich genommen haben, 1 Getränk entspricht z.B. 0,125l Wein oder 0,33l Bier oder 4cl Spirituosen.[A]	Anzahl der Getränke ___	dose
Wie intensiv war das Erlebnis?	Gar nicht ⊢——⊣ So intensiv[P] [energiegeladen, aufgedreht[C] / alkoholisiert, betrunken[A]] wie nur vorstellbar	intensity
Ich fühlte mich besonders durchsetzungsfähig.	Nein, nicht mehr als gewöhnlich ⊢——⊣ Ja, ich erlebte dies komplett/vollständig	eii1
Ich erlebte eine Auflösung meines „Selbst“ oder Ego.	Nein, nicht mehr als gewöhnlich ⊢——⊣ Ja, ich erlebte dies komplett/vollständig	edi1
Ich fühlte mich wichtiger oder außergewöhnlicher als andere.	Nein, nicht mehr als gewöhnlich ⊢——⊣ Ja, ich erlebte dies komplett/vollständig	eii2
Ich fühlte mich eins mit dem Universum.	Nein, nicht mehr als gewöhnlich ⊢——⊣ Ja, ich erlebte dies komplett/vollständig	edi2
Mein Ego fühlte sich aufgeblasen an.	Nein, nicht mehr als gewöhnlich ⊢——⊣ Ja, ich erlebte dies komplett/vollständig	eii3
Ich empfand ein Gefühl von Einigkeit mit anderen.	Nein, nicht mehr als gewöhnlich ⊢——⊣ Ja, ich erlebte dies komplett/vollständig	edi3
Ich fühlte mich meiner selbst besonders sicher.	Nein, nicht mehr als gewöhnlich ⊢——⊣ Ja, ich erlebte dies komplett/vollständig	eii4
Ich erlebte meine eigene Wichtigkeit als vermindert.	Nein, nicht mehr als gewöhnlich ⊢——⊣ Ja, ich erlebte dies komplett/vollständig	edi4
Ich fühlte mich besonders erpicht und wetteifernd.	Nein, nicht mehr als gewöhnlich ⊢——⊣ Ja, ich erlebte dies komplett/vollständig	eii5
Ich erlebte einen Zerfall meines „Selbst“ oder Ego.	Nein, nicht mehr als gewöhnlich ⊢——⊣ Ja, ich erlebte dies komplett/vollständig	edi5
Ich empfand meine Sicht als mehr wert als die anderer Leute.	Nein, nicht mehr als gewöhnlich ⊢——⊣ Ja, ich erlebte dies komplett/vollständig	eii6
Ich fühlte mich weit weniger von meinen Sorgen und Problemen vereinnahmt.	Nein, nicht mehr als gewöhnlich ⊢——⊣ Ja, ich erlebte dies komplett/vollständig	edi6
Ich fühlte mich besonders selbstbewusst.	Nein, nicht mehr als gewöhnlich ⊢——⊣ Ja, ich erlebte dies komplett/vollständig	eii7

Ich verlor jegliches Ichgefühl.	Nein, nicht mehr als gewöhnlich	Ja, ich erlebte dies komplett/vollständig	edi7
Ich fühlte mich besonders selbstsicher.	Nein, nicht mehr als gewöhnlich	Ja, ich erlebte dies komplett/vollständig	eii8
Jeglicher Begriff von Selbst und Identität zerfloss.	Nein, nicht mehr als gewöhnlich	Ja, ich erlebte dies komplett/vollständig	edi8
Alle Dinge schienen sich zu einem einzigen Ganzen zu vereinen.[P]	Nein, nicht mehr als gewöhnlich	Ja, ich erlebte dies komplett/vollständig	5dasc18
Ich fühlte mich körperlos.[P]	Nein, nicht mehr als gewöhnlich	Ja, ich erlebte dies komplett/vollständig	5dasc26
Ich war unfähig, auch nur die kleinste Entscheidung zu treffen.[P]	Nein, nicht mehr als gewöhnlich	Ja, ich erlebte dies komplett/vollständig	5dasc27
Ich hatte Angst, aus meinem Zustand nicht mehr herauszukommen.[P]	Nein, nicht mehr als gewöhnlich	Ja, ich erlebte dies komplett/vollständig	5dasc32
Ich fühlte mich eins mit meiner Umgebung.[P]	Nein, nicht mehr als gewöhnlich	Ja, ich erlebte dies komplett/vollständig	5dasc34
Sorgen und Ängste des Alltags kamen mir belanglos vor.[P]	Nein, nicht mehr als gewöhnlich	Ja, ich erlebte dies komplett/vollständig	5dasc35
Ich verspürte einen Hauch von Ewigkeit.[P]	Nein, nicht mehr als gewöhnlich	Ja, ich erlebte dies komplett/vollständig	5dasc41
Gegensätze und Widersprüche schienen sich aufzulösen.[P]	Nein, nicht mehr als gewöhnlich	Ja, ich erlebte dies komplett/vollständig	5dasc42
Ich hatte Angst, ohne genau sagen zu können weshalb.[P]	Nein, nicht mehr als gewöhnlich	Ja, ich erlebte dies komplett/vollständig	5dasc43
Ich erlebte alles beängstigend verzerrt.[P]	Nein, nicht mehr als gewöhnlich	Ja, ich erlebte dies komplett/vollständig	5dasc44
Meine Umgebung kam mir fremd und unheimlich vor.[P]	Nein, nicht mehr als gewöhnlich	Ja, ich erlebte dies komplett/vollständig	5dasc46
Ich fühlte mich wie gelähmt[P]	Nein, nicht mehr als gewöhnlich	Ja, ich erlebte dies komplett/vollständig	5dasc47
Vergangenheit, Gegenwart und Zukunft erlebte ich als eine Einheit.[P]	Nein, nicht mehr als gewöhnlich	Ja, ich erlebte dies komplett/vollständig	5dasc52
Ich hatte das Gefühl einer unerträglichen Leere.[P]	Nein, nicht mehr als gewöhnlich	Ja, ich erlebte dies komplett/vollständig	5dasc53
Ich fühlte mich bedroht.[P]	Nein, nicht mehr als gewöhnlich	Ja, ich erlebte dies komplett/vollständig	5dasc56

Mein Körper erschien mir gefühllos, leblos, fremd.[P]	Nein, nicht mehr als gewöhnlich ⊢—⊣ Ja, ich erlebte dies komplett/vollständig	5dasc60
Ich hatte das Gefühl, außerhalb meines Körpers zu sein.[P]	Nein, nicht mehr als gewöhnlich ⊢—⊣ Ja, ich erlebte dies komplett/vollständig	5dasc62
Ich fühlte mich, als ob ich schweben würde.[P]	Nein, nicht mehr als gewöhnlich ⊢—⊣ Ja, ich erlebte dies komplett/vollständig	5dasc63
Ich fühlte mich isoliert von allem und jedem.[P]	Nein, nicht mehr als gewöhnlich ⊢—⊣ Ja, ich erlebte dies komplett/vollständig	5dasc64
Die Grenze zwischen mir und meiner Umgebung schien sich zu verwischen.[P]	Nein, nicht mehr als gewöhnlich ⊢—⊣ Ja, ich erlebte dies komplett/vollständig	5dasc71
Ich hatte das Gefühl, keinen eigenen Willen mehr zu haben.[P]	Nein, nicht mehr als gewöhnlich ⊢—⊣ Ja, ich erlebte dies komplett/vollständig	5dasc78
Ich hatte Angst, die Kontrolle über mich zu verlieren.[P]	Nein, nicht mehr als gewöhnlich ⊢—⊣ Ja, ich erlebte dies komplett/vollständig	5dasc79
Ich empfand ein Gefühl der Ehrfurcht.[P]	Nein, nicht mehr als gewöhnlich ⊢—⊣ Ja, ich erlebte dies komplett/vollständig	5dasc81
Ich hatte das Gefühl, es würde Schreckliches geschehen.	Nein, nicht mehr als gewöhnlich ⊢—⊣ Ja, ich erlebte dies komplett/vollständig	5dasc89
Ich empfand eine allumfassende Liebe.[P]	Nein, nicht mehr als gewöhnlich ⊢—⊣ Ja, ich erlebte dies komplett/vollständig	5dasc91
Freiheit aus der Begrenzung der eigenen Persönlichkeit und Gefühl der Einheit mit etwas, das größer war als Sie selbst.[P]	Kein(e), gar nicht<br>So schwach, kann ich nicht genau sagen<br>Schwach<br>Mittel<br>Stark<br>Extrem (mehr als jemals zuvor in meinem Leben)	meq14
Gefühl der Einheit in Verbindung mit einer inneren Welt.[P]	Kein(e), gar nicht<br>So schwach, kann ich nicht genau sagen<br>Schwach<br>Mittel<br>Stark<br>Extrem (mehr als jemals zuvor in meinem Leben)	meq20
Erfahrung des Eintretens der eigenen Person in ein größeres Ganzes.[P]	Kein(e), gar nicht<br>So schwach, kann ich nicht genau sagen<br>Schwach<br>Mittel<br>Stark<br>Extrem (mehr als jemals zuvor in meinem Leben)	meq26
Erfahrung der Einheit mit einer Letzten Wirklichkeit.[P]	Kein(e), gar nicht<br>So schwach, kann ich nicht genau sagen<br>Schwach<br>Mittel<br>Stark<br>Extrem (mehr als jemals zuvor in meinem Leben)	meq28

Gefühl, dass Sie Ewigkeit oder Unendlichkeit erlebten.[P]	Kein(e), gar nicht<br>So schwach, kann ich nicht genau sagen<br>Schwach<br>Mittel<br>Stark<br>Extrem (mehr als jemals zuvor in meinem Leben)	meq5
Gefühl der Einheit mit Objekten und/oder Personen, die Sie in Ihrer Umgebung wahrnahmen.[P]	Kein(e), gar nicht<br>So schwach, kann ich nicht genau sagen<br>Schwach<br>Mittel<br>Stark<br>Extrem (mehr als jemals zuvor in meinem Leben)	meq6
Erfahrung der Einsicht, dass „alles Eins ist".[P]	Kein(e), gar nicht<br>So schwach, kann ich nicht genau sagen<br>Schwach<br>Mittel<br>Stark<br>Extrem (mehr als jemals zuvor in meinem Leben)	meq18
Erfahrung des reinen Seins oder des reinen Bewusstseins.[P]	Kein(e), gar nicht<br>So schwach, kann ich nicht genau sagen<br>Schwach<br>Mittel<br>Stark<br>Extrem (mehr als jemals zuvor in meinem Leben)	meq16
Bewusstsein für das Leben oder die Lebendigkeit in allen Dingen.[P]	Kein(e), gar nicht<br>So schwach, kann ich nicht genau sagen<br>Schwach<br>Mittel<br>Stark<br>Extrem (mehr als jemals zuvor in meinem Leben)	meq25
Mein Körper endete an der Grenze zwischen meiner Haut und der Umwelt.[P]	-100 — 100<br>Ich fühlte meinen Körper weit über die Grenzen meiner Haut erweitert.	pci11
Ich bewahrte die ganze Zeit über eine sehr starke Bewusstheit meiner selbst.[P]	-100 — 100<br>Ich bewahrte überhaupt keine starke Bewusstheit meiner selbst.	pci50
Ich behielt ständig ein starkes Gefühl der Trennung zwischen mir und der Umgebung.[P]	-100 — 100<br>Ich erfuhr eine intensive Einheit mit der Welt; die Grenzen zwischen mir und der Umgebung lösten sich auf.	pci51
Meine Körpergefühle schienen sich in die mich umgebende Welt auszudehnen.[P]	-100 — 100<br>Meine Körpergefühle waren auf den Bereich innerhalb meiner Haut begrenzt.	pci26
Wie bedeutungsvoll war das Erlebnis für Sie persönlich?	Nicht mehr als gewöhnliche, alltägliche Erlebnisse<br>Vergleichbar mit bedeutungsvollen Erlebnissen, die sich durchschnittlich einmal pro Woche ereignen<br>Vergleichbar mit bedeutungsvollen Erlebnissen, die sich durchschnittlich einmal pro Monat ereignen<br>Vergleichbar mit bedeutungsvollen Erlebnissen, die sich durchschnittlich einmal pro Jahr ereignen<br>Vergleichbar mit bedeutungsvollen Erlebnissen, die sich durchschnittlich einmal alle 5 Jahren ereignen<br>Unter den 10 bedeutungsvollsten Erlebnissen meines Lebens<br>Unter den 5 bedeutungsvollsten Erlebnissen meines Lebens<br>Das bedeutungsvollste Erlebnis meines Lebens	how_meaningful

Bitte geben Sie an, inwieweit das Erlebnis für Sie spirituell bedeutsam war.	Kann ich nicht sagen<br>Gar nicht<br>Schwach<br>Mittel<br>Stark<br>Unter den 5 spirituell bedeutsamsten Erlebnissen meines Lebens<br>Das spirituell bedeutsamste Erlebnis meines Lebens	how_spiritual
Glauben Sie, dass das Erlebnis und die Verarbeitung des Erlebnisses eine Veränderung Ihres derzeitigen persönlichen Wohlbefindens oder Ihrer Lebenszufriedenheit bewirkt hat?	Sehr stark vermindert<br>Mäßig vermindert<br>Schwach vermindert<br>Keine Veränderung<br>Schwach gesteigert<br>Mäßig gesteigert<br>Sehr stark gesteigert	change_wellbeing
Haben Sie abschließend Kommentare, Anmerkungen oder Ergänzungen zu Ihren speziellen Erlebnissen?	Freitextfeld	comment1
Haben Sie abschließend Kommentare, Anmerkungen oder Ergänzungen zu dieser Umfrage?	Freitextfeld	comment2
